# Antiretroviral Drugs Impact Autophagy: Opportunities for Drug Repurposing

**DOI:** 10.31083/j.fbl2907242

**Published:** 2024-07-02

**Authors:** Laura Cheney, John M. Barbaro, Grace McDermott, Joan W. Berman

**Affiliations:** 1 Department of Medicine, Division of Infectious Diseases, Albert Einstein College of Medicine, Bronx, NY 10461, USA; 2 Department of Pathology, Albert Einstein College of Medicine, Bronx, NY 10461, USA; 3 Albert Einstein College of Medicine, Bronx, NY 10461, USA; 4 Department of Microbiology and Immunology, Albert Einstein College of Medicine, Bronx, NY 10461, USA

**Keywords:** autophagy, LC3, p62, age-related diseases, cancer, chemotherapy, cell death, antiretroviral drug, HIV, drug repositioning

## Abstract

Autophagy is an evolutionarily conserved process in which intracellular macromolecules are degraded in a lysosomal-dependent manner. It is central to cellular energy homeostasis and to quality control of intracellular components. A decline in autophagic activity is associated with aging, and contributes to the development of various age-associated pathologies, including cancer. There is an ongoing need to develop chemotherapeutic agents to improve morbidity and mortality for those diagnosed with cancer, as well as to decrease the cost of cancer care. Autophagic programs are altered in cancer cells to support survival in genetically and metabolically unstable environments, making autophagy an attractive target for new chemotherapy. Antiretroviral drugs, which have dramatically increased the life- and health spans of people with human immunodeficiency virus (HIV) (PWH), have offered promise in the treatment of cancer. One mechanism underlying the antineoplastic effects of antiretroviral drugs is the alteration of cancer cell autophagy that can potentiate cell death. Antiretroviral drugs could be repurposed into the cancer chemotherapy arsenal. A more complete understanding of the impact of antiretroviral drugs on autophagy is essential for effective repurposing. This review summarizes our knowledge of the effects of antiretroviral drugs on autophagy as potential adjunctive chemotherapeutic agents, and highlights gaps to be addressed to reposition antiretroviral drugs into the antineoplastic arsenal successfully.

## 1 Introduction

### 1.1 Autophagy

**Fig. 1.**
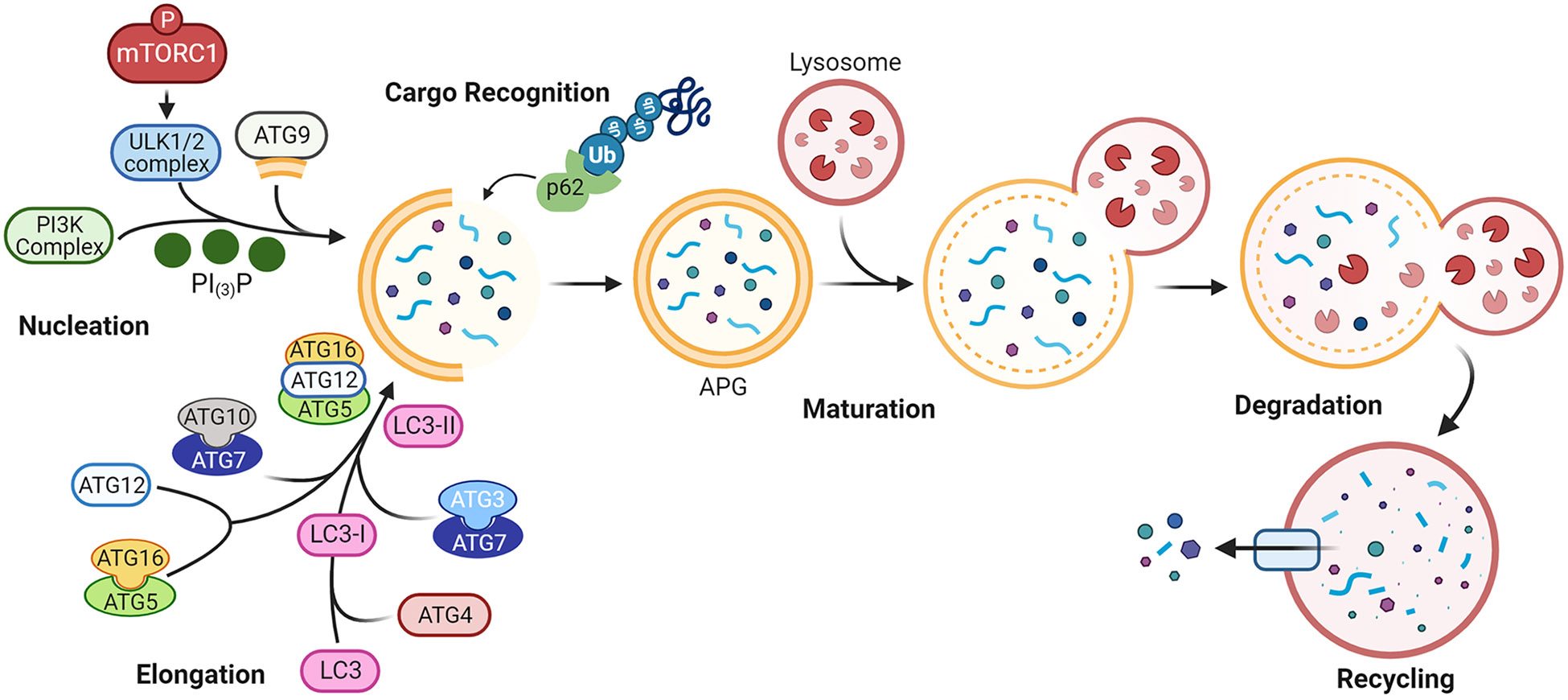
Schematic of macroautophagy. More than 30 proteins are involved in this dynamic process. Autophagy is initiated upon phosphorylation of mTORC1, inactivating the complex with which it associates. This enables a pre-initiation complex that includes UNC-51 like kinase family proteins (ULK1/2), to recruit a class III phosphatidylinositol 3-kinase (PI3K) complex. Phosphatidylinositol-3-phosphate (PI_(3)_P) then increases at intracellular membrane sites, recruiting ATG9 which transports pieces of double membrane to nucleate a nascent autophagosome (APG). A ubiquitin-conjugation-like complex, ATG7-ATG10/ATG3 activates a second ubiquitin-conjugation-like complex, ATG5-ATG12. This complex, in concert with other autophagy-related (ATG) proteins, processes and lipidates microtubule-associated protein 1A/1B light chain 3B (LC3) into LC3-II. LC3-II is a component of both the inner and outer membranes of the APG. It facilitates APG elongation and closure, and participates in cargo recognition. P62 also aids in cargo recognition, transporting polyubiquitinated proteins to the forming APG for incorporation inside. Once the APG double membrane encloses, Rab family GTPases and SNARE superfamily proteins (not pictured) mediate fusion of the APG outer membrane with a lysosome, in a process called maturation. Cargo degradation then ensues, including that of the LC3-II that was present on the inner APG membrane. LC3-II on the outer membrane can be converted back to LC3-I to be used again in APG biogenesis.

Autophagy is a highly conserved proteolytic process that removes damaged and toxic macromolecules, protein aggregates, organelles, and infectious pathogens from the intracellular environment. There are three major types of autophagy: macroautophagy, microautophagy, and chaperone-mediated autophagy. Macroautophagy, henceforward termed autophagy, is the major type discussed in this review. In macroautophagy, intracellular substrates, or cargo, are incorporated inside a double membrane vesicle, called an autophagosome (APG), that fuses with a lysosome for cargo degradation, as detailed in Fig. 1. Briefly, an appropriate stimulus leads to phosphorylation of mTORC1, resulting in a series of phosphorylation events, protein complex recruitment, and phosphatidylinositol signaling that culminates in a new double membraned APG [1]. Cargo is incorporated inside the APG as its membrane is elongated. A fully formed APG fuses with a lysosome, and cargo inside is degraded [1]. The products of this degradation can be exported out of the lysosome to fulfill needs of the cell, such as production of energy substrates, macromolecules, and organelles. The protein microtubule-associated protein 1A/1B light chain 3B (LC3)-II, hereafter called LC3-II, associates with APG from nucleation to degradation, and is used commonly as a marker for assessing autophagy dynamics. APG can enclose around cytosolic cargo non-specifically, or cargo can be targeted to APG specifically, termed selective autophagy. Selective autophagy is mediated by autophagy receptors and adapters, such as p62 (Fig. 1). These contain protein binding domains that recognize degradation signals on cargo for cargo selection as well as LC3 interacting regions that facilitate cargo recruitment into forming APG. p62, which binds mostly ubiquitinated proteins, is degraded within the lysosome similarly to LC3-II, and as such, is another common marker used to assess autophagy.

Autophagy is constitutive, and is also dynamic, increasing in response to homeostatic imbalance. While the first recognized purpose of autophagy was protection from nutrient-deprivation, it is now well-recognized to participate in many cell processes, including various modes of cell death. Extensive evidence from genetic and pharmacologic models across species demonstrates that alterations in autophagic activity can drive cell, tissue, and organism dysfunction, thus negatively impacting health- and lifespan, including contributing to aging and the pathogenesis of age-related diseases such as cancer. The relationship between autophagy and tumorigenesis is extraordinarily complex. Broadly, autophagy can both suppress tumorigenesis as well as promote tumor progression. Once a tumorigenic clone has emerged, autophagic processes support cancer cell metabolism, increase cancer cell resistance to inhospitable conditions such as hypoxia, reactive oxygen species (ROS), limited nutrients and trophic factors, and anticancer therapeutics, and can undermine the immune response to cancer cells [2,3]. Altering autophagy in cancer cells can result in cancer cell death [4-6]. This makes modulation of autophagy an appealing target for chemotherapy.

### 1.2 Chemotherapy and Drug Repurposing

Cancer is a leading cause of death in adults worldwide, second only to heart diseases [7]. Approximately 40% of U.S. men and women will be diagnosed with any type of cancer at some point in their lifetime [8], although advancing age is the single greatest risk factor for cancer development, with approximately 80% of all cancers being diagnosed in people aged 55 years or greater [8].

Cancer death rates in the U.S. decreased on average by 2.3% per year in men, and 1.9% per year in women from 2015 to 2019 [9]. There were almost 17 million cancer survivors in 2019, and this is projected to increase to 22.2 million by 2030 [8]. These rates are afforded by everimproving cancer detection methods and treatment modalities. Despite these positive trends, there are 2 million new cancer diagnoses, and over 600,000 deaths projected in the U.S. alone for 2023 [8]. The national patient economic burden associated with cancer care in 2019 was over $21 billion [8], and as the population both ages and grows, and as new chemotherapeutic agents are developed, the cost of cancer care is expected to increase.

There is an ongoing need to develop therapies to improve morbidity and mortality, as well as cost of care, for people diagnosed with cancer and undergoing treatment. An understanding of the cellular processes that lead to cancer as well as the development of drug resistance underpins the advancement of novel and effective chemotherapeutic agents. Dysregulation of many cell processes leads to cancerous transformation. Autophagy is one such process, and altering autophagy in cancer cells can lead to cancer cell death [2-6]. Autophagy is therefore an attractive target for chemotherapy drug development. However, discovery and testing of novel agents, regardless of whether they target autophagy, requires significant time (years) and cost (billions).

Drug repurposing, or drug repositioning, is a strategy by which new uses for approved drugs are identified; these uses are distinct from that of the original purpose of the drug. There are many advantages of drug repurposing, including reduced time frames to authenticate a new indication, because the safety and formulations of a repurposed drug are already established. Reduced time frames translate to faster access to efficacious treatments, as well as to decreased research and development costs. A detailed review of these concepts and more can be found in [10-12].

Antiretroviral drugs, developed for treatment of infection with Human Immunodeficiency Virus (HIV), have shown promise in the treatment of a variety of cancers. Their antineoplastic effect is distinct from antiretroviral activity. There are several mechanisms that underlie their antitumor actions, including modulation of autophagy. Because of the importance of autophagy to cancer cell survival, the impacts of antiretroviral drugs on autophagy may be exploited to reposition them as adjunctive chemotherapeutic agents.

### 1.3 Antiretroviral Drugs

Amazing strides have been made in the management of HIV infection since the beginning of the HIV epidemic 40 years ago. Prior to antiretroviral therapy (ART), infection with HIV conferred high morbidity and swift mortality. As a result of the greater than 35 different antiretroviral drugs that have been developed to treat HIV (Table 1), people with HIV (PWH) now have significantly improved quality and quantity of life.

PWH who begin and maintain an ART regimen have a rapid reduction in viral load, steady T cell recovery both in naïve and memory cell compartments, and eventually, sustained immune reconstitution. Associated with immune restoration is a decreased incidence of Acquired Immunodeficiency Syndrome (AIDS)-associated malignancies, particularly Kaposi’s Sarcoma, Non-Hodgkin’s lymphomas, and anogenital tumors, tumor regression, prolonged time to treatment failure, and longer survival. The major attribution to these findings is a return of immunologic control of the viruses, Human Herpesvirus-8, Epstein-Barr Virus, and Human Papilloma Virus, that are associated with these malignancies, respectively. However, several studies have shown that the anti-tumor effects of antiretroviral drugs are not well correlated with markers of immune reconstitution, suggesting there are additional anti-tumor mechanisms. Many antiretroviral drugs have pleiotropic antitumor effects. They block telomerase activity, inhibit the proteosome, Akt signaling, matrix metalloproteases, and angiogenesis, and may alter epitope processing to modulate antitumor immune responses, and impact autophagy, among other mechanisms. These impacts are important for optimizing antiretroviral repositioning, and have been reviewed elsewhere. To facilitate research of anti-cancer mechanisms, examples of these effects are in Supplementary Table 1, and we refer the reader to the following reviews: [13-17].

It is known that antiretroviral drugs impact autophagy [18]. However, knowledge of these impacts is limited in the context of cancer. A comprehensive understanding of the effects of antiretrovirals specifically on autophagy in the context of cancer will help guide their repositioning as antineoplastics. In this review, we focus on changes in autophagy mediated by antiretroviral drugs of different classes in various cell types to contribute to the arrest of cancer cell proliferation or to cause cell death (Table 2, Ref. [19-52]). The ultimate goal is to improve not just the clinical outcomes for people with cancer by overcoming chemotherapy resistance or chemotherapy failure through autophagy modulation, but also decrease the financial burden of cancer care by repurposing antiretroviral drugs.

## 2. Protease Inhibitors

Protease inhibitors (PI) (Table 1) were introduced in 1995, and remain an important component of modern ART regimens. PI inhibit the viral aspartyl protease, preventing the processing of viral polyproteins into functional forms that comprise mature virions. Concomitant with widespread use of PI, the incidence of AIDS-associated malignancies decreased [13,17]. This led to great interest in evaluating PI for repurposing into antineoplastic drugs. There are numerous activities of PI that lead to anti-cancer effects (Supplementary Table 1), including modulating autophagy.

### 2.1 Nelfinavir

Among the PI, Nelfinavir (NFV) appears to be the most potent and broadly acting, with effects on angiogenesis, cell invasion, AKT signaling, and apoptosis [17]. NFV also causes significant endoplasmic reticulum (ER) stress [17]. Cells respond to ER stress, in part, by upregulating autophagy. This adaptive response is cytoprotective by reducing the toxicity of accumulated misfolded proteins and protein aggregates. Understanding a cell’s autophagic response to NFV increases knowledge of NFV effects that could be used for chemotherapy.

NFV appears to induce autophagy in several different cancer cell models. Autophagy was induced in H157 cells, a human non-small cell lung cancer (NSCLC) cell line, treated with 10 or 20 μM NFV. There was increased LC3-II protein, increased LC3 puncta by fluorescence microscopy, and vacuoles were noted on transmission electron microscopy (TEM) [19]. LC3-II protein was also increased in H157 xenografts in mice treated with NFV [19], suggesting effects on autophagy occur *in vivo* as well as *in vitro*. In another study, autophagy was induced in TT and MZ-CRC-1 cells, lines used in models of medullary thyroid cancer. LC3-II protein was increased and p62 protein was decreased after treatment with 10 μM NFV [20]. Similarly, autophagy was induced in the human prostate cancer cell lines DU145 and PC-3 [21]. These cells, transfected to express GFP-LC3, had decreased GFP fluorescence by flow cytometry after treatment with 20 μM NFV. This result was similar to Rapamycin, an inducer of autophagic flux. In another study, NFV treatment of primary human chronic lymphocytic leukemia (CLL) cells also induced autophagy. Five μM and 10 μM NFV increased LC3 puncta by immunofluorescence, and decreased p62 protein, similarly to Rapamycin [22]. Lastly, autophagy was induced in mouse embryonic fibroblasts (MEF) knocked out for Tuberous Sclerosis Complex 2 (*TSC2^−/−^*) and treated with 20 μM NFV. Loss of TSC2 causes excess mTOR signaling that inhibits autophagy. Mutations causing loss of TSC2 function are present in a small percentage of bladder and pancreatic neuroendocrine cancers [53,54]. NFV treatment increased LC3-II and decreased p62 protein in *TSC2^−/−^* MEF, indicating increased autophagic flux [23]. For reference, the maximum concentration (C_max_) of NFV in serum is 3–10 μM [55].

NFV does not induce autophagy in every cancer cell type; rather, it may inhibit autophagy in some cell types. This was demonstrated in a study of multiple myeloma [24]. Bortezomib, a proteosome inhibitor used in the treatment of multiple myeloma, induced the colocalization of LC3 and Lysosome Associated Membrane Protein 2 (LAMP2), a lysosome marker, in NCI-H929 cells by immunofluorescent microscopy [24]. This effect was mitigated by concomitant treatment with 5 μM NFV. The authors conclude that autophagy was upregulated as a cytoprotective response to Bortezomib stress, and NFV impaired APG maturation, i.e., inhibited autophagy. In another study of ME-180 cells, a cervical cancer cell line, there was increased LC3-II and p62 protein by Western blotting, as well as increased LC3 and p62 puncta by immunofluorescence after 10 μM NFV treatment [25]. The authors conclude that autophagy was induced. While chloroquine (CQ) was used as a positive control for autophagy inhibition, flux analyses were not performed. An alternative interpretation is that with accumulation of both LC3-II and p62 as evidenced by two distinct techniques, NFV inhibited autophagy.

Multiple other studies attempt to link the potential chemotherapeutic efficacy of NFV to effects on autophagy but the true effects of NFV on autophagy are difficult to determine based on the studies performed. For example, in a study of four different breast cancer cells lines, T47D, MCF-7, MDA-MB-453, and MDA-MB-435 [26], 26 μM NFV increased LC3-II protein by Western blotting in MDA-MB-453 cells only. The same authors performed a separate study with MDA-MB-453 cells, and found a time-dependent increase in LC3-II protein with the same NFV concentration [27]. In a separate breast cancer model using the MCF-7 breast cancer cell line, treatment with increasing concentrations of NFV (max 6.67 μM) increased LC3-I protein by Western blotting [28]. In another breast cancer model, there was a time-dependent increase in LC3-II after treatment of MDA-MB-231 cells with 25 μM NFV plus 20 μM 2,5-Dimethyl-celecoxib, an ER stress inducer [29]. In a study of non-small cell lung cancer, different concentrations of NFV, 7.5 μM and 25 μM, were packaged into nanoparticles that were used to treat A549 cells, a lung cancer cell line. There was a dose-dependent increase in LC3B protein by Western blotting [30]. In a cervical cancer model using SiHa and HeLa cells, treatment with a combination of 4 μM NFV plus 10 μM Metformin, a widely used type II diabetes medication, led to an increase of APG by TEM, a decrease in the LC3-I/LC3-II ratio, and ATG3, Beclin-1, and ATG7 protein by Western blotting [31]. In a model of pediatric leukemia, neither 10 nor 20 μM NFV treatment of SEM and Molm13 cells, acute lymphocytic leukemia and acute myelocytic leukemia cell lines, respectively, changed LC3-II protein amount by Western blotting [32]. Similarly, no change in LC3-II was detected by Western blotting after 10 μM NFV treatment of H157 or A549 cells in a human lung cancer model [33], nor after 10 μM NFV treatment of CAOV3 cells in an ovarian cancer model [34]. Lastly, in a phase I/II clinical trial of 29 patients with refractory Multiple Myeloma, LC3-II was increased in Peripheral Blood Mononuclear Cells (PBMC) from the patients after 16 weeks of treatment with NFV plus both lenalidomide, an anti-angiogenic agent, and dexamethasone [35]. While the authors of these studies conclude that autophagy was induced in the models, flux assays were not performed to differentiate autophagy induction from inhibition of APG maturation, analysis of LC3-I as opposed to LC3-II was performed, analysis of LC3 without differentiating between LC3-I or LC3-II was performed, or NFV was not studied independently of other drugs. Some studies do have similar findings of increased LC3-II. It is therefore tempting to examine the totality of the data across studies and conclude that NFV consistently induces autophagy. However, it is uncertain that this conclusion can be drawn based on different NFV concentrations, cell lines, and techniques used to measure autophagy.

### 2.2 Saquinavir

Saquinavir (SQV) was the first protease inhibitor approved for treatment of HIV, and was important to the development of combination antiretroviral therapy. One group examined the antineoplastic effect of SQV on ten different ovarian cancer cells lines [36]. They found a dose- and time-dependent inhibition of cell growth and apoptosis with SQV treatment in both chemo-sensitive (A2780) and chemo-resistant (SKOV3 and CAOV3) lines. To characterize the mechanism underlying cell death, they examined markers of ER stress, ATF6 and GRP78, and examined autophagy by presence of APG by electron microscopy, and formation of GFP-LC3 puncta in GFP-LC3 transfected cells after treatment with 50 μM SQV. They found increased ATF6 and GRP78 suggestive of ER stress, and presence of APG by EM and an increase in GFP-LC3 puncta. The authors conclude that ER stress induced autophagy leading to cell death, and therefore SQV may have clinical application in the treatment of ovarian cancer. While cell death did occur at lower SQV concentrations, ER stress and autophagy studies were performed with a high dose of SQV. Ritonavir-boosted SQV serum C_max_ ranges from only 2.1 to 26 μM [56]. Another limitation to the study is that flux assays were not performed to confirm that increased LC3 puncta was a result of induced autophagy rather than inhibited maturation.

In another study of ovarian cancer, the effects of SQV on cisplatin resistance, which confers a poor prognosis, was determined [37]. SKOV3 cells were treated with 10 or 20 μM SQV, and the concentration of cisplatin required to cause 50% cell death, or IC50, was measured [37]. The IC50 of cisplatin increased with SQV, suggesting that SQV caused an increase in cisplatin resistance. Inhibiting autophagy with 3-methyladenine (3-MA) during SQV treatment restored cisplatin IC_50_ to control level, suggesting autophagy plays a role in cisplatin resistance. The authors found that SQV increased both mTOR and BECN1 RNA and protein, concluding that SQV increased autophagy, thereby promoting cisplatin resistance [37]. While of interest, mTOR and BECN1 have opposing effects on autophagy, and no additional techniques were performed to assess autophagy directly. More work is needed to clarify the contribution of SQV in this context.

Infection with Human Papillomavirus strain 16 (HPV16) increases the risk for development of oropharyngeal and anogenital cancers. HIV PI were shown to decrease cervical dysplasia in cancer and precursor lesions in women with HIV. HPV16 has two oncoproteins, E6 and E7, whose constitutive expression is required to maintain HPV16-positive cancers. One group examined the effects of PI on HPV16 E6/E7 [38]. They used a model of pre-cancerous HPV16 infection: normal immortalized keratinocytes carrying 10-50 extrachromosomal copies of HPVP16 (NIKS16). NIKS16 cells form organoid rafts similar to human skin and maintain infection with HPV16 throughout cell differentiation stages. NIKS16 rafts treated with 5 μM SQV were atrophic, and had decreased cell proliferation relative to control [38]. The authors could not detect E6/E7 by immunofluorescence of the rafts, but they did find MCM7, a protein expressed in the presence of E7. SQV decreased the number of MCM7-positive cells in the rafts, suggesting SQV decreased E7 [38]. E6/E7 inhibit autophagosome fusion with the lysosome, an important step for HPV16 cancer development. The authors examined LC3 and p62 by immunofluorescence of the rafts, and found a small decrease of LC3 and a significant decrease of p62. They conclude that SQV reversed E6/E7 autophagy inhibition. While they did not determine whether the shift in autophagy dynamics by SQV contributed to decreased E6/E7 oncoprotein level, reversal of HPV16 autophagy inhibition is likely important for decreasing HPV16 oncogenic potential.

### 2.3 Lopinavir

Lopinavir (LPV) is a unique PI in that it was not formulated for use independently from Ritonavir (RTV, discussed below) because it has poor bioavailability. One group identified Lopinavir as a potential chemotherapeutic agent through its effects on autophagy in a model of metastatic gastric cancer [39]. When non-cancerous cells detach from the extracellular matrix (ECM), they undergo a type of programmed cell death termed anoikis. Cancer cells detaching from the ECM and intravasating into the vasculature must be anoikis resistant to survive and establish metastatic disease. In certain prostate and liver cancer models, autophagy was shown to promote metastasis by inhibiting anoikis. ATG4B, which is essential for the lipidation of LC3 (Fig. 1), is expressed highly in various cancer types. Inhibiting ATG4B may induce anoikis, thus inhibiting metastatic processes, but ATG4B inhibitors are not currently clinically viable options. Using AGS cells, a human gastric cancer cell line, the authors demonstrate that high levels of ELAVL1, an RNA-binding protein, suppress *circSPECC1* levels, a specific sequence of circular RNA, which binds to and negatively regulates ATG4B [39]. The effect of ELAVL1 on *circSPECC1* led to increased ATG4B levels, thereby increasing autophagy as shown by LC3 and p62 Western blotting and fluorescence microscopy of an mCherry-GFP-LC3 reporter. This interaction conferred anoikis resistance to the cells. Molecular docking and virtual screening studies identified LPV as a molecule that could disrupt the interaction between ELAVL1 and *circSPECC1*. After treatment with 25 μM or 50 μM LPV, *circSPECC1* levels were restored, and correspondingly, ATG4B levels and autophagy were decreased. LPV increased anoikis *in vitro*, and decreased the incidence of pulmonary metastasis after tail vein injection of GC in a nude mouse model [39]. This study introduces LPV as a viable chemotherapeutic agent for metastatic gastric cancer. One limitation of the study is the concentration of LPV used. The serum C_max_ of LPV is 21 μM when dosed twice a day with RTV [57].

Having determined that a nitric oxide (NO)-releasing derivative of SQV had anti-cancer properties, one group generated a similar molecule from Lopinavir (LPV-NO) to examine its efficacy in glioblastoma and melanoma models in two separate studies [40,41]. LN-229 and U-251 cells, human glioblastoma cell lines [40], and B16, B16F, mouse melanoma cells lines, and A375 cells, a human melanoma cell line [41], were treated with a range of LPV and LPV-NO concentrations. In both models, the IC_50_ of LPV-NO was confirmed to be lower than that of LPV, and it reduced cell viability, decreased cell proliferation, and induced differentiation of cells into more terminal phenotypes. To assess changes in autophagy, LPV-NO-treated cells were stained with acridine orange, which labels only late-stage autolysosomes and cannot provide information on autophagy dynamics. In all cell types, acridine orange staining was increased after LPV-NO treatment. However, neither cell viability or differentiation was rescued when cells were treated with 3-MA [40,41]. In the glioblastoma model, LPV-NO improved cell viability and decreased apoptosis in cells co-treated with Cisplatin relative to cells treated with Cisplatin alone [40]. This was correlated with an increase in acridine orange staining. The authors conclude that autophagy was upregulated as a cytoprotective response to LPV-NO induced oxidative stress, and could be a mechanism by which resistance to Cisplatin develops. While LPV-NO appeared to be beneficial in these models, the role of autophagy remains unclear as 3-MA did not impact cell viability or differentiation, and only the acridine orange staining was performed.

### 2.3 Indinavir

Indinavir has been suggested to block angiogenesis and tumor cell invasion, but not inhibit proliferation or induce apoptosis in cancer cells *in vitro* [58]. To improve Indinavir’s antineoplastic properties, one group developed an analog, CH05-10 [42]. Human lung cancer cells, A549, were treated with CH05-10, and apoptosis, ER stress, and autophagy were assessed. CH05-10 decreased cell viability, inhibited proliferation, caused G1 cell cycle arrest, and increased apoptosis in a concentration- and time-dependent manner. It also caused ER stress, as Binding Immunoglobulin Protein and C/EBP homologous protein (CHOP) proteins were increased, as well as spliced X-box binding protein 1 (*XBP-1*) mRNA [42]. LC3-II was increased after CH05-10 treatment, and cell death was enhanced after inhibition of autophagy with 3-MA. They conclude that autophagy upregulation is a cytoprotective response to the ER stress caused by CH05-10 [42]. With pleiotropic effects, this analog is a promising chemotherapeutic candidate, although more autophagy-directed studies are needed to understand fully its effects on autophagy.

### 2.4 Ritonavir

Originally used as treatment in PWH to inhibit HIV protease, Ritonavir (RTV) is now used in current regimens to increase, or boost serum levels of other protease inhibitors. This is because RTV is a potent inhibitor of Cytochrome P450 (CYP3A). One group examined its possible efficacy in treating glioblastoma [43]. Patient-derived glioblastoma cells (pGBM) were treated with 30 μM RTV. The mean serum C_max_ of treatment-dose RTV is 15 μM [59]. Treated cells underwent cell cycle arrest, doubling time increased almost two-fold, and there was decreased migration with decreased gene expression of matrix metalloproteases. These findings were augmented when cells were concomitantly treated with temozolomide (TMZ), a standard chemotherapeutic agent for glioblastoma. To identify the mechanisms underlying the increased efficacy of combined RTV and TMZ, the authors examined ER stress and autophagy in pGBM treated with 15 μM RTV with or without TMZ. They found increased markers of ER stress (BiP, XBP1 and ATF4), increased LC3-II by Western blotting, and accumulation of vacuoles associated with autophagic flux with a commercially available dye kit. Addition of 4-phenylbutyric acid (4-PBA), which abolishes ER stress by inhibiting aggregation of misfolded proteins, to cells treated with RTV+TMZ increased the metabolic activity of pGBM relative to RTV+TMX treated cells without 4-PBA, suggesting ER stress and autophagy are important mediators of cancer cell compromise in their model [43]. The authors conclude that RTV induced autophagy; however, flux assays were not performed to ensure that the increased LC3-II and vacuoles on imaging were not due to inhibition of autophagic flux.

### 2.5 Darunavir

Darunavir is the first-line preferred protease inhibitor in ART regimens [60]. As for all PI in modern regimens, Darunavir is boosted with either low-dose RTV, or with cobicistat, a non-antiretroviral inhibitor of Cytochrome P450 (CYP3A). Precursor analogs of Darunavir, RDD-19 and RDD-142, which had been developed originally for potential use as antiretrovirals, were tested for efficacy as antitumor agents against hepatocellular carcinoma [44]. While Darunavir itself did not cause any cytotoxicity, RDD-19 and RDD-142 reduced viability and induced apoptosis in HepG2 cells, a hepatocellular carcinoma cell line, in a dose-dependent manner. These findings were associated with a dose-dependent increase of ER stress markers as well as inhibition of the proteosome. They also performed Western blotting for LC3 and Beclin-1, and found significantly increased amounts of LC3-II relative to control, but no change in Beclin-1. Additional studies were not performed to further evaluate the effects of the compounds on autophagy. The authors conclude that while the IC_50_ of either RDD-19 or RDD-142 were suboptimal, both are good lead compounds for further development into anticancer agents by their pleiotropic effects on the cancer cell line [44].

## 3. Reverse Transcriptase Inhibitors

The first FDA approved medication to treat HIV infection was a reverse transcriptase (RT) inhibitor (RTI). While RTI inhibit viral reverse transcriptase with different mechanisms, they all cause chain termination during viral replication (Table 1). These remain the mainstay of HIV treatment [60]. Similar to PI, the incidence of cancers in PWH decreased with the institution of RTI in HIV treatment regimens [61,62]. As was also found for PI, RTI may mediate chemotherapeutic effects by several mechanisms (Supplementary Table 1), including autophagy modulation.

### 3.1 Efavirenz

Efavirenz (EFV) is a non-nucleoside reverse transcriptase inhibitor that was an important component of ART regimens for almost two decades. Many studies showed that EFV can inhibit cell proliferation and promote cell death in various carcinoma and sarcoma cell lines [63-69], and a small clinical trial showed EFV may have some efficacy in treating metastatic castration-resistant prostate cancer [70]. In a recent publication, 20 μM EFV and 20 μM SPV122.2, a stereoisomer of EFV, induced significant DNA damage, chromatin reorganization, lamin B1 breakdown with micronuclei formation, and ultimately, decreased proliferation and significant apoptosis of PC3 cells, a prostate carcinoma cell line [45]. As LC3-II plays a role in lamin B1 degradation [71,72], the authors examined whether autophagy contributes to EFV/SPV122.2 induced nuclear changes. They saw increased LC3 positive puncta by immunofluorescent microscopy, as well as increased LC3-II, p62, and ATG7 by Western blotting. While these studies do not exclude the possibility that APG maturation was inhibited, pharmacologic inhibition of autophagy with 3-MA mitigated the effects of EFV and SPV122.2 on nuclear architecture, and restored viability of the malignant cells, suggesting EFV and SPV122.2 induced autophagy which contributed to the effects on the nucleus [45]. Daily dosing of EFV leads to an average serum level range between 3.17 and 12.67 μM [73]. Due to major interpersonal EFV pharmacokinetic variability, 20–40% of people can have plasma levels that reach as high as 30–50 μM [74-76]. Their data suggest that activating autophagy by EFV may have chemotherapeutic benefit in prostate cancer.

In a study of colon cancer, EFV was used in combination with Metformin and Fluoxetine, a common antidepressant, to generate excess ROS as a means to induce cancer cell death [46]. There was a significant increase in ROS production in HCT116 cells, a colon cancer cell line, after treatment with 1.5 μM EFV plus Metformin and Fluoxetine relative to any of the three drugs alone. This combination also reduced mitochondrial membrane potential, induced cell cycle arrest, and caused significant cell death. To identify the underlying mechanisms of toxicity, Western blotting was performed for markers of DNA damage, apoptosis, necroptosis, and of p62 to assess autophagy. Most markers were increased relative to control, including p62. However, the increase in p62 was small and not statistically significant. They conclude that this drug combination had profound antitumor effects due to ROS induced DNA damage, and upregulation of apoptosis, necroptosis and autophagy. However, an increase in p62 suggests autophagy may be inhibited. In addition, excess p62 stabilizes nuclear factor erythroid 2-related factor 2 (NRF2) [77], a major transcription factor involved in the defense against oxidative and electrophilic stresses. *SQSTM1*, the gene for p62, is a target of NRF2. [78]. The increased p62 may be a result of a positive feed-back loop established with activation of the NRF2 anti-oxidant system rather than resulting from a change in autophagy. As there were no direct studies of autophagy, it remains unclear what effect EFV or the 3-drug combination may have on autophagy in this model.

### 3.2 Dapivirine

Dapivirine is a unique antiretroviral, developed as an active ingredient in microbicides and vaginal rings to prevent HIV acquisition in women. One study showed Dapivirine to have potential therapeutic benefit in a glioblastoma model [47]. U87 cells, a human glioblastoma cell line, were treated with a range of Dapivirine concentrations, and cell viability and apoptosis were examined. Dapivirine decreased cell proliferation, and increased apoptosis with an IC_50_ of 10.73 μM. They assessed autophagy after treatment with 16 μM, and found a time-dependent increase in the LC3-II/I ratio, and time-dependent fluctuations in ATG7 and BCN-1. They also examined the antineoplastic activity of 16 mg/kg subcutaneous Dapivirine in mice that developed tumors after transplantation of U87 cells. Tumor volumes were significantly decreased, and apoptosis in the tumor tissue was also increased in the Dapivirine treated mice. The authors conclude that stress induced by Dapivirine leads to autophagy induction, but this fails to effectively protect the stressed cells, resulting in apoptosis [47]. Use of LC3-II/I as the sole determinant of autophagy activity is controversial [79]. In addition, there was no apparent change in LC3-II levels compared to control cells, and they report finding no changes in autophagy related proteins in the tumor tissue (data not shown). The anticancer effects of Dapivirine seem promising, but more studies are needed to understand the effects of Dapivirine on autophagy more completely.

### 3.3 Zalcitabine

Zalcitabine (ddC) did not achieve great success in the treatment of HIV as it was inconveniently dosed, less potent than other NRTI, and had overlapping toxicities with RTI, limiting its ability to be used in combination with other RTI. One study examined the effect of ddC on mitochondrial function and autophagy in a pancreatic cancer model. Treatment of two human pancreatic ductal adenocarcinoma (PDAC) cell lines, PANC1 and Capan2, and of *ex vivo* primary human cells with 20 μM ddC led to significant mitochondrial dysfunction with reduced mtDNA copy number, increased ROS, decreased oxygen consumption, and reduced ATP production. ddC also induced cell death [48]. mtDNA replication requires Transcriptional Factor A, Mitochondrial (TFAM), which can also activate the DNA damage sensing pathway. ddC treatment reduced TFAM, and increased cytosolic mtDNA, ultimately leading to increased phosphorylation of Stimulator of Interferon Response CGAMP Interactor 1 (STING1) [48], a cytosolic DNA sensor linked to the innate immune response and autophagy. The authors used a novel autophagic flux reporter, GFP-LC3-RFP-LC3∆G [80], to show that ddC increases autophagic flux in a STING1-dependent manner, and that increased autophagy leads to ferroptosis [48], an iron-dependent form of autophagy-regulated cell death [81]. Their study increases understanding of the crosstalk between the DNA damage response and autophagy, and supports manipulating autophagy to treat pancreatic cancer. For reference, serum C_max_ of ddC ranges from 0.055 μM to 0.170 μM [82].

### 3.4 Abacavir

The guanosine analog Abacavir (ABC) was once a preferred backbone agent for PWH, and is still a first-line preferred and alternative agent for children with HIV [60]. One study examined ABC as a potential chemotherapeutic agent for medulloblastoma (MB) [49], a common pediatric brain tumor with a low 5-year survival rate. MB is thought to arise, in part, from abnormal neuronal or glial cell differentiation. ABC had been previously shown to induce cell differentiation [83]. The authors used ABC in an attempt to induce cell differentiation and reduce cancerforming capacity. Three human medulloblastoma cell lines, DAOY, MEB-Med8a, and D238-Med, were treated with 250 μM, 40 μM, and 240 μM ABC, respectively, after which they found significantly more double-strand DNA breaks, reduced cell viability, increased apoptosis, and decreased clonogenic survival. This was associated with a significant increase in the “autophagy activity factor (AAF)” which they derived from the mean fluorescence intensities of treated and control cells using a commercially available dye kit for measuring autophagic flux. The authors conclude that the increased AAF is evidence of increased autophagy [49], but it is not clear if the appropriate controls were used in order to analyze autophagic flux properly. In addition, the concentrations of ABC used represent an inhibitory concentration by which 90% of clonogenic cells were killed (IC_90_) in preliminary experiments. The average serum and CSF concentrations of ABC are 4.86 μM and 4.47 μM, respectively [84]. Further work is needed to understand more fully the effects of ABC on autophagy, as a possible adjunct chemotherapeutic agent for medulloblastoma.

### 3.5 Tenofovir

Tenofovir is a NRTI (Table 1) and a major component of contemporary ART, being one of two NRTI recommended in first- and second line regimens. It is also a component of Pre-exposure Prophylaxis (PrEP) as well as Pose-Exposure Prophylaxis (PEP), preventing HIV infection in individuals at risk for HIV acquisition. It was recently examined as a possible chemotherapeutic agent for breast cancer [50]. Breast cancer was induced in Sprague Dawley rats with 7,12-dimethylbenz(a) anthracene (DMBA) given twice weekly for four weeks. During this four-week period, rats were also given 25 mg/kg Tenofovir (TF25), 50 mg/kg Tenofovir (TF50), or Doxorubicin (DOX)+TF50. TF25 and TF50 conferred a survival advantage, and mammary gland tissue weight was significantly decreased after all three treatments. Markers of oxidative stress, cell proliferation, and apoptosis were all decreased after the three treatments. Enzyme-linked immunosorbent assay (ELISA) for Beclin-1 and LC3 were performed on supernatants obtained from homogenized breast tissue, and the three treatments decreased Beclin-1, and increased LC3 relative to DMBA alone. The authors conclude that TF offers therapeutic benefit, even more so when combined with DOX, as a result of decreasing breast tissue weight, decreasing oxidative stress, apoptosis and cell proliferation, as well as increasing autophagy [50]. However, the ELISA measured total LC3, and no other autophagy analysis was performed. Tenofovir may be beneficial in the treatment of breast cancer, but the effects on autophagy are not fully characterized.

### 3.6 Etravirine

Etravirine (ETR) is a NNRTI (Table 1) that has a flexible conformation, enabling it to bind RT effectively even in the presence of RT mutations. At the time of its FDA approval, ETR offered HIV treatment efficacy for those in whom resistance to other NNRTI had developed. ETR was shown to have anti-cancer effects in an ovarian cancer model, possibly by impacting autophagic degradation of Anterior gradient protein 2 homolog (AGR2) [51]. AGR2, thought to play a role in protein folding, is expressed highly in many cancers, promoting angiogenesis, enhancing metastasis and tumor progression. Its expression is associated with chemoresistance and a poor prognosis. The authors treated two ovarian cancer cell lines, A2780 and A2780-ADR, with ETR ranging from 1.25 μM to 10 μM, and performed LC3-II Western blotting. They found a dose-dependent increase in LC3-II, corresponding to a dose-dependent decrease of AGR2. By immunofluorescence, LC3 was increased after 10 μM ETR and this also corresponded with decreased AGR2. When autophagy was inhibited with wortmannin, a PI3K inhibitor, CQ, or ATG7 siRNA knockdown, the effects of ETR in AGR2 were reversed, suggesting that ETR decreased AGR2 level by autophagy-mediated degradation. ETR also decreased cell proliferation, viability and migration *in vitro*, and when combined with Paclitaxel, a common chemotherapy agent, decreased cancer progression and metastasis in a xenograft mouse model. The authors conclude that ETR is a promising chemotherapeutic agent for its ability to upregulate autophagic degradation of AGR2 [51]. Of note, the median serum C_max_ of ETR is 1.3 μM [85]. While the authors show an effect on LC3-II with a biologically relevant concentration of ETR, the majority of the autophagy studies were performed with higher concentrations.

## 4. Entry Inhibitors

Entry Inhibitors (Table 1) were important to the evolution of successful ART regimens, as they offered a novel mechanism of action against HIV in treatment-experienced patients in whom resistance to PI and RTI had developed. Members of this class of medications have diverse mechanisms of actions, all preventing HIV from entering cells. They too have shown potential to act as chemotherapeutic agents (Supplementary Table 1). One group studied how endothelial cells contribute to prostate cancer metastasis, incorporating Maraviroc into their animal model [52]. Maraviroc is a C-C chemokine receptor type 5 (CCR5) antagonist, blocking HIV gp120 from binding CCR5, one of two cell surface co-receptors needed for HIV to enter a cell. Using a coculture of C4-2 cells, a metastatic prostate cancer cell line, and human umbilical vein endothelial cells (HU-VEC), the authors show that HUVEC enhanced prostate cancer cell invasion by decreasing androgen receptor (AR) activity in a C-C chemokine ligand type 5 (CCL5)- dependent manner. AR signaling inhibits autophagy, which degrades paxillin, an important component of focal adhesion sites where cells anchor their actin cytoskeletons to the ECM. Paxillin accumulates during autophagy inhibition, which can increase cell mobility. In their model, CCL5 repression of AR led to increased autophagy (as evidenced by increased LC3-II and decreased p62 on Western blotting), reduced paxillin, and subsequently increased cell migration. When either a CCL5 neutralization antibody or CQ was added to the co-culture, autophagy was inhibited (as shown by accumulation of LC3-II on Western blotting), paxillin was increased, and cell mobilization was decreased. In their mouse model in which prostate cancer was induced by injection of C4-2 plus HUVEC cells, mice treated with either Maraviroc or CQ had decreased metastatic foci compared to control mice, and mice treated with both Maraviroc and CQ had no metastatic disease. This supported their conclusion that CCL5, CCLR5 and/or autophagy are potential drug targets to inhibit endothelial cell promotion of prostate cancer metastases [52].

## 5. Discussion and Conclusions

Autophagy is required for maintaining cell, and by extension, tissue and organism homeostasis. A decline in or dysregulation of autophagic activity contributes to aging, as well as to age-dependent pathologies, including cancer. Tumorigenesis and autophagy have a complex and incompletely understood relationship, but their interrelationship affords great opportunity for chemotherapy development. Repurposing antiretroviral drugs would be a more time efficient and less costly mechanism to bring “new” chemotherapeutic agents to market. The positive effects of antiretroviral drugs on cancer cells in *in vitro* and pre-clinical studies have led to many clinical trials of antiretrovirals to reposition them as adjunctive chemotherapeutic agents in people with a variety of malignancies (Supplementary Table 2). The impacts of antiretroviral drugs on autophagy likely contribute to their anti-cancer properties.

The studies reviewed highlight that antiretroviral drugs have varied, cell-specific effects on autophagy (Table 2). Effects on autophagy are not antiretroviral drug class specific, nor is there one specific effect by individual drugs. NFV best underscores this point, as NFV can induce [19-23], inhibit [24,25], or have no effect [32-34] on autophagy depending on the model examined. The choice of antiretroviral drug to be used as part of a chemotherapy regimen will need to be tailored to the specific cancer depending on the desired effect on autophagy of the antiretroviral drug. To provide clinical benefit through autophagy modulation, the effect on autophagy would need to potentiate cytotoxicity or cell death. While many studies demonstrate increased cell death after antiretroviral drug treatment [19-21,23-25,36,42,45,47-49], not all studies link changes in autophagy to cell death [25-27,47,49], nor did all antiretroviral drugs induce cell death despite possibly altering autophagy [20,22,24,28,37]. For example, SQV increased the IC_50_ of cisplatin in an ovarian cancer model, and LPV-NO decreased Cisplatin efficacy in a lung cancer model [37,40], and while the effects on autophagy are unclear, increasing cisplatin resistance is an undesirable effect. Increased knowledge of the specific impacts of antiretroviral drugs on autophagy in the context of cell death could guide repositioning to optimize therapeutic benefit. While the effects of antiretroviral drugs on autophagy in some models cannot be determined as they were not studied in isolation [29,31,35], combination studies are important to continue as antiretrovirals are likely to fill an adjuvant role in cancer treatment. The number of options for chemotherapeutic regimens could increase with additional knowledge of the effects of antiretroviral drugs in combination with antineoplastic agents. An additional avenue for drug development includes modifying existing antiretroviral compounds to improve potency, as demonstrated with CH05-10 [42], LPV-NO [40,41], RDD-19 and RDD-142 [44], and SPV122.2 [45]. Several studies demonstrate additive or synergistic effects of antiretroviral drugs on cell death when combined with pharmacologic inhibitors of autophagy. For example, apoptosis was augmented when NFV or CH05-10 treatment was concomitant with 3-MA [19,42], or when NFV or Maraviroc was given with CQ [20-22,52]. These studies highlight that inhibition of the cytoprotective autophagic response to acute cell stress when induced by antiretroviral drugs is a viable chemotherapeutic strategy.

There are several potential mechanisms underlying the effects of antiretroviral drugs on autophagy. ER stress is a well-known inducer of autophagy. Autophagy is upregulated to mitigate damage from toxic, misfolded proteins and protein aggregates. A few studies show increased markers of ER stress with clear evidence of autophagy induction after NFV treatment [19,22,23]. Other studies demonstrate presence of ER stress after antiretroviral treatment [26-29,32,36,42-44,50], and while data may suggest autophagy was induced in these models, it is unclear whether autophagy is consistently induced with ER stress [86]. The specific molecular factors involved in the ER stress response and downstream effects on autophagy and apoptosis in the presence of antiretroviral drugs are important to identify for possible chemotherapeutic potential. Other mechanisms by which antiretroviral drugs may alter autophagy include mitochondrial damage and dysfunction [20,46,48], DNA damage and nuclear structural abnormalities [23,45,46,49], oxidative stress [26,31,50], and possibly calpain inhibition [24].

There are two major limitations to many studies reviewed herein. First, autophagic flux assays or multiple complimentary autophagy assays were frequently not performed to assess autophagy dynamics. While there are no fixed criteria for determining the status of autophagy in every experimental context, there are regularly updated guidelines that detail acceptable assays as well provide as an extensive framework with which to interpret experimental results [79]. Autophagy is a highly dynamic, multistep process that can be positively and/or negatively impacted at several steps. It is important to perform techniques that allow for the distinction of changes in specific steps in autophagy. For example, the accumulation of GFP-LC3 puncta by fluorescent microscopy represents either increased APG formation, or inhibition of APG maturation. The experimental result is the same, yet the step in autophagy that is affected is different. The effect of autophagy on cell death and ultimately on human diseases mandates the use of precise tools such as in [39,48] to measure and correctly interpret autophagic flux changes in response to stimuli, especially in the context of repositioning antiretroviral drugs as cancer treatment. Second, the concentrations of antiretroviral drugs used in many studies exceeds achievable human serum or CSF concentrations, at least with the current antiretroviral formulations. Some antiretroviral drugs have considerable variability in steady-state serum concentrations both inter- and intra-individually, however, use of high drug concentrations in *in vitro* studies may lead to detection of artifact. Drugs should be used in concentrations reflective of serum or CSF levels, depending on the tissue of interest, to model human pharmacokinetics and pharmacodynamics better. In addition, an antiretroviral drug progressing through the chemotherapy pipeline based on data generated from concentrations exceeding current formulations would have to undergo reformulation and new clinical safety trials.

A single molecular pathway does not contribute solely to either aging or to cancer biology. There are several hallmarks of each, many of which overlap, including autophagy [2]. Decreased autophagic activity is highly correlated with both aging and tumor progression. As autophagy contributes to many cell processes including mitochondrial and other organelle homeostasis, ROS neutralization, degradation of toxic aggregates, and metabolism, age-associated decline in autophagy likely dysregulates these processes too, thereby contributing to cancer development. It is not known to what degree these other cell processes are impacted by autophagy in the context of aging, nor the extent of their contributions to tumorigenesis. Also, age-related changes to autophagy may promote cancer development in both cell-autonomous and non-autonomous manners. Autophagy likely shapes the microenvironment of tissue as much as it does the intracellular environment of cells. There is currently little understanding of the impacts of non-autonomous age-related autophagy changes to cancer cells and the development or progression thereof. This review has addressed only macroautophagy, but the other forms of autophagy, such as chaperone-mediated autophagy, also decrease with advancing age [87,88]. As knowledge related to macroautophagy in the context of aging and cancer increases, understanding of other types of autophagy and how they intersect with macroautophagy, cancer, and aging should also expand. Research opportunities, and thus cancer treatment opportunities, will continue to increase when effects of antiretroviral drugs are examined within all of these contexts. From the clinical and drug development perspective, antiretrovirals provide a research advantage over novel molecules because the safety profiles of antiretroviral drugs in aging persons have been determined.

As the population grows and ages, the need for new chemotherapy agents will continue to increase. Repurposing approved drugs that could effect cancer cell death or increase chemosensitivity by autophagy modulation is both time-saving and cost-effective. A more complete understanding of the effects of antiretroviral drugs on autophagy in cancer models will increase the opportunity to reposition antiretrovirals into the antineoplastic arsenal, positively impacting the morbidity and mortality, as well as the financial burden of care for people with cancer.

## Supplementary Material

supplementary tables

## Figures and Tables

**Table 1.** Antiretroviral drugs by class

Entry Inhibitor^1^	Fusion Inhibitor^2^	Casid Inhibitor^2^	Rev. Transc. Inhibitors^4^	Integrase Inhibitors^5^	Protease Inhibitors^6^
Maraviroc Ibalizumab Fostemsavir Cenicriviroc^α^	Enfuvirtide	Lenacapavir	NRTI^β^	Raltegravir Elvitegravir Dolutegravir Bictegravir Cabotegravir	Saquinavir Indinavir Nelfinavir Ritonavir Lopinavir Amprenavir Atazanavir Fosamprenavir Tipranavir Darunavir
			Zidovudine Didanosine Zalcitabine Stavudine Lamivudine Abacavir Tenofovir Emtricitabine Dapivirine		
			NNRTI^γ^		
			Nevirapine Delavirdine Efavirenz Etravirine Rilpivirine Doravirine		
			NRTTI^δ^		
			Islatravir^α^		

1 Entry Inhibitors prevent viral infection of cells by preventing binding of viral proteins to host cell surface receptors. Each have different mechanisms of action

α Investigational—not currently FDA approved.

2 Binds to HIV glycoprotein (gp) 41 to prevent its conformational change required for the fusion of viral and cellular membranes

3 Binds to the hexamers of viral capsid p24 protein, disrupting the capsid, which is needed in multiple steps of the viral life cycle

4 Reverse Transcriptase (RT) Inhibitors (RTI) inhibit viral reverse transcriptase:

β Nucleo(t)side RTI are analogs of naturally occurring deoxynucleotides except they cannot form the next 5–3’ phosphodiester bond with an incoming nucleotide

γ Non-nucleotide RTI bind directly to RT, preventing movement of protein domains needed to carry out viral DNA synthesis

δ Nucleoside RT Translocation Inhibitor, is a new class of RTI that prevent opening of the RT binding site such that new nucleotides cannot be incorporated.

5 Integrase Strand Transfer Inhibitors (ISTI) prevent integration of HIV DNA into host chromosomal DNA.

6 Protease Inhibitors (PI) inhibit HIV aspartyl protease from processing viral polyproteins into individual functional proteins.

**Table 2.** Summary of studies that examine effects of antiretroviral drugs on autophagy in the context of cancer.

Antiretroviral drug	Malignancy	Cell type/Model	Effect on autophagy^1^	Ref.
Protease Inhibitors				
Nelfinavir	Non-small cell lung	H157, AnNCr-nu/nu mice	Increased autophagy	[19]
	Medullary Thyroid	TT, MZ-CRC-1	Increased autophagy	[20]
	Prostate	DU145, PC-3	Increased autophagy	[21]
	CLL^3^	Primary human CLL	Increased autophagy	[22]
	Bladder, pancreas	Mouse embryonic fibroblasts	Increased autophagy	[23]
	Multiple Myeloma	NCI-H929	Decreased LC3/LAMP2 colocalization	[24]
	Cervical	ME-180	Increased LC3-II, p62 protein, puncta	[25]
	Breast	MDA-MB-453	Increased LC3-II	[26]
	Breast	MDA-MB-453	Increased LC3-II	[27]
	Breast	MCF-7	Increased LC3-I	[28]
+2,5-dimethyl-celecoxib	Breast	MDA-MB-231	Increased LC3-II	[29]
	Non-small cell lung	A549	Increased LC3B	[30]
+metformin	Cervical	SiHa, HeLa	Increased APG, decreased LC3-I/LC3-II	[31]
	Pediatric ALL^4^, AML^5^	SEM, Molm13	No change in LC3-II	[32]
	Non-small cell lung	H157, A549	No change in LC3-II	[33]
	Ovarian	CAOV3	No change in LC3-II	[34]
+lenalid., dexameth.^2^	Multiple Myeloma	Primary Human PBMC^6^	Increased LC3-II	[35]
Saquinavir	Ovarian	A2780, SKOV3, CAOV3	APG on EM, increased LC3 puncta	[36]
	Ovarian	SKOV3	Increased mTOR, BECN1 RNA, protein	[37]
	Cervical	NIKS16	Decreased LC3, p62 puncta	[38]
Lopinavir	Gastric	AGS	Decreased autophagy	[39]
Lopinavir analog	Glioblastoma	LN-229, U-251	Increased late-stage autolysosomes	[40]
	Melanoma	B16, B16F10, A375	Increased late-stage autolysosomes	[41]
Indinavir analog	Lung	A549	Increased LC3-II	[42]
Ritonavir	Glioblastoma	pGBC^7^	Increased LC3-II, vacuoles	[43]
Two Darunavir analogs	Hepatocellular	HepG2	Increased LC3-II	[44]
Rev. Transc. Inhibitors				
Efavirenz	Prostate	PC3, LNCaP, PNT2	Increased autophagy	[45]
+metformin, fluoxetine	Colon	HCT116	Increased p62	[46]
Dapivirine	Glioblastoma	U87	Increased LC3-II/I ratio	[47]
Zalcitabine	Pancreas	PANC1, Capan2	Increased autophagy	[48]
Abacavir	Medulloblastoma	DAOY, MEB-Med8a, D283-Med	Increased “autophagy activity factor”	[49]
Tenofovir	Breast	DMBA^8^ Sprague Dawley rats	Increased LC3, Beclin-1	[50]
Etravirine	Ovarian	A2780, A2780-ADR	Increased autophagy	[51]
Entry Inhibitors				
Maraviroc	Prostate	C4-2 injected BALB/c-nude mice	Decreased autophagy	[52]

1 Major findings are reported when multiple assays or autophagic flux assays were not performed

2 Lenalidomide, dexamethasone

3 Chronic Lymphocytic Leukemia

4 Acute Lymphocytic Leukemia

5 Acute Myelocytic Leukemia

6 Peripheral Blood Mononuclear Cells

7 Patient derived glioblastoma cells

8 7,12-dimethylbenz(a) anthracene

## References

[1] Bento CF, Renna M, Ghislat G, Puri C, Ashkenazi A, Vicinanza M, Mammalian Autophagy: How Does It Work? Annual Review of Biochemistry. 2016; 85: 685–713. 10.1146/annurev-biochem-060815-014556 26865532

[2] López-Otín C, Pietrocola F, Roiz-Valle D, Galluzzi L, Kroemer G. Meta-hallmarks of aging and cancer. Cell Metabolism. 2023; 35: 12–35. 36599298 10.1016/j.cmet.2022.11.001

[3] Cassidy LD, Narita M. Autophagy at the intersection of aging, senescence, and cancer. Molecular Oncology. 2022; 16: 3259–3275. 35689420 10.1002/1878-0261.13269 PMC9490138

[4] Mathew R, Karantza-Wadsworth V, White E. Role of autophagy in cancer. Nature Reviews. Cancer 2007; 7: 961–967. 17972889 10.1038/nrc2254 PMC2866167

[5] Li X, He S, Ma B. Autophagy and autophagy-related proteins in cancer. Molecular Cancer. 2020; 19: 12. 31969156 10.1186/s12943-020-1138-4 PMC6975070

[6] Bhat P, Kriel J, Shubha Priya B, Basappa, Shivananju NS, Loos B. Modulating autophagy in cancer therapy: Advancements and challenges for cancer cell death sensitization. Biochemical Pharmacology. 2018; 147: 170–182. 29203368 10.1016/j.bcp.2017.11.021

[7] Ferlay J, Ervik M, Lam F, Laversanne M, Colombet M, Mery L, 2024; Global Cancer Observatory: Cancer Today. Available at: https://gco.iarc.who.int/today (Accessed: 15 November 2023).

[8] SEER*Explorer: An interactive website for SEER cancer statistics [Internet]. Surveillance Research Program, National Cancer Institute; Data source(s): SEER Incidence Data, November 2023 Submission (1975-2021), SEER 22 registries. 2024. Available at: https://seer.cancer.gov/statistics-network/explorer/ (Accessed: 8 June 2023).

[9] Henley SJ, Ward EM, Scott S, Ma J, Anderson RN, Firth AU, Annual report to the nation on the status of cancer, part I: National cancer statistics. Cancer. 2020; 126: 2225–2249. 32162336 10.1002/cncr.32802 PMC7299151

[10] Pushpakom S, Iorio F, Eyers PA, Escott KJ, Hopper S, Wells A, Drug repurposing: progress, challenges and recommendations. Nature Reviews. Drug Discovery. 2019; 18: 41–58. 30310233 10.1038/nrd.2018.168

[11] Ajmeera D, Ajumeera R. Drug repurposing: A novel strategy to target cancer stem cells and therapeutic resistance. Genes & Diseases. 2023; 11: 148–175. 37588226 10.1016/j.gendis.2022.12.013 PMC10425757

[12] Siddiqui S, Deshmukh AJ, Mudaliar P, Nalawade AJ, Iyer D, Aich J. Drug repurposing: re-inventing therapies for cancer without re-entering the development pipeline-a review. Journal of the Egyptian National Cancer Institute. 2022; 34: 33. 35934727 10.1186/s43046-022-00137-0 PMC9358112

[13] Monini P, Sgadari C, Toschi E, Barillari G, Ensoli B. Antitumour effects of antiretroviral therapy. Nature Reviews. Cancer 2004; 4: 861–875. 15516959 10.1038/nrc1479

[14] Chow WA, Jiang C, Guan M. Anti-HIV drugs for cancer therapeutics: back to the future? The Lancet. Oncology 2009; 10: 61–71. 19111246 10.1016/S1470-2045(08)70334-6

[15] Pfab C, Schnobrich L, Eldnasoury S, Gessner A, El-Najjar N. Repurposing of Antimicrobial Agents for Cancer Therapy: What Do We Know? Cancers. 2021; 13: 3193. 34206772 10.3390/cancers13133193 PMC8269327

[16] Koltai T. Nelfinavir and other protease inhibitors in cancer: mechanisms involved in anticancer activity. F1000Research. 2015; 4: 9. 26097685 10.12688/f1000research.5827.1 PMC4457118

[17] Gantt S, Casper C, Ambinder RF. Insights into the broad cellular effects of nelfinavir and the HIV protease inhibitors supporting their role in cancer treatment and prevention. Current Opinion in Oncology. 2013; 25: 495–502. 23872785 10.1097/CCO.0b013e328363dfee PMC4029099

[18] Cheney L, Barbaro JM, Berman JW. Antiretroviral Drugs Impact Autophagy with Toxic Outcomes. Cells. 2021; 10: 909. 33920955 10.3390/cells10040909 PMC8071244

[19] Gills JJ, Lopiccolo J, Tsurutani J, Shoemaker RH, Best CJM, Abu-Asab MS, Nelfinavir, A lead HIV protease inhibitor, is a broad-spectrum, anticancer agent that induces endoplasmic reticulum stress, autophagy, and apoptosis in vitro and in vivo. Clinical Cancer Research: an Official Journal of the American Association for Cancer Research. 2007; 13: 5183–5194. 17785575 10.1158/1078-0432.CCR-07-0161

[20] Kushchayeva Y, Jensen K, Recupero A, Costello J, Patel A, Klubo-Gwiezdzinska J, The HIV protease inhibitor nelfinavir down-regulates RET signaling and induces apoptosis in medullary thyroid cancer cells. The Journal of Clinical Endocrinology and Metabolism. 2014; 99: E734–E745. 24483157 10.1210/jc.2013-3369

[21] Guan M, Fousek K, Chow WA. Nelfinavir inhibits regulated intramembrane proteolysis of sterol regulatory element binding protein-1 and activating transcription factor 6 in castration-resistant prostate cancer. The FEBS Journal. 2012; 279: 2399–2411. 22540830 10.1111/j.1742-4658.2012.08619.x

[22] Mahoney E, Maddocks K, Flynn J, Jones J, Cole SL, Zhang X, Identification of endoplasmic reticulum stress-inducing agents by antagonizing autophagy: a new potential strategy for identification of anti-cancer therapeutics in B-cell malignancies. Leukemia & Lymphoma. 2013; 54: 2685–2692. 23469959 10.3109/10428194.2013.781168 PMC3815958

[23] Johnson CE, Hunt DK, Wiltshire M, Herbert TP, Sampson JR, Errington RJ, Endoplasmic reticulum stress and cell death in mTORC1-overactive cells is induced by nelfinavir and enhanced by chloroquine. Molecular Oncology. 2015; 9: 675–688. 25498902 10.1016/j.molonc.2014.11.005 PMC5528710

[24] Escalante AM, McGrath RT, Karolak MR, Dorr RT, Lynch RM, Landowski TH. Preventing the autophagic survival response by inhibition of calpain enhances the cytotoxic activity of bortezomib in vitro and in vivo. Cancer Chemotherapy and Pharmacology. 2013; 71: 1567–1576. 23572175 10.1007/s00280-013-2156-3 PMC3669633

[25] Davis MA, Delaney JR, Patel CB, Storgard R, Stupack DG. Nelfinavir is effective against human cervical cancer cells in vivo: a potential treatment modality in resource-limited settings. Drug Design, Development and Therapy. 2016; 10: 1837–1846. 27330277 10.2147/DDDT.S102241 PMC4898046

[26] Brüning A, Friese K, Burges A, Mylonas I. Tamoxifen enhances the cytotoxic effects of nelfinavir in breast cancer cells. Breast Cancer Research: BCR. 2010; 12: R45. 20594311 10.1186/bcr2602 PMC2949632

[27] Brüning A, Rahmeh M, Friese K. Nelfinavir and bortezomib inhibit mTOR activity via ATF4-mediated sestrin-2 regulation. Molecular Oncology. 2013; 7: 1012–1018. 23916134 10.1016/j.molonc.2013.07.010 PMC5528439

[28] Chakravarty G, Mathur A, Mallade P, Gerlach S, Willis J, Datta A, Nelfinavir targets multiple drug resistance mechanisms to increase the efficacy of doxorubicin in MCF-7/Dox breast cancer cells. Biochimie. 2016; 124: 53–64. 26844637 10.1016/j.biochi.2016.01.014

[29] Thomas S, Sharma N, Golden EB, Cho H, Agarwal P, Gaffney KJ, Preferential killing of triple-negative breast cancer cells *in vitro* and *in vivo* when pharmacological aggravators of endoplasmic reticulum stress are combined with autophagy inhibitors. Cancer Letters. 2012; 325: 63–71. 22664238 10.1016/j.canlet.2012.05.030

[30] Parvathaneni V, Goyal M, Kulkarni NS, Shukla SK, Gupta V. Nanotechnology Based Repositioning of an Anti-Viral Drug for Non-Small Cell Lung Cancer (NSCLC). Pharmaceutical Research. 2020; 37: 123. 32514688 10.1007/s11095-020-02848-2

[31] Xia C, He Z, Liang S, Chen R, Xu W, Yang J, Metformin combined with nelfinavir induces SIRT3/mROS-dependent autophagy in human cervical cancer cells and xenograft in nude mice. European Journal of Pharmacology. 2019; 848: 62–69. 30695683 10.1016/j.ejphar.2019.01.045

[32] Meier-Stephenson V, Riemer J, Narendran A. The HIV protease inhibitor, nelfinavir, as a novel therapeutic approach for the treatment of refractory pediatric leukemia. OncoTargets and Therapy. 2017; 10: 2581–2593. 28553123 10.2147/OTT.S136484 PMC5440076

[33] Lopiccolo J, Kawabata S, Gills JJ, Dennis PA. Combining Nelfinavir With Chloroquine Inhibits *In Vivo* Growth of Human Lung Cancer Xenograft Tumors. In Vivo (Athens, Greece). 2021; 35: 141–145. 33402459 10.21873/invivo.12241 PMC7880743

[34] Bowers RR, Andrade MF, Jones CM, White-Gilbertson S, Voelkel-Johnson C, Delaney JR. Autophagy modulating therapeutics inhibit ovarian cancer colony generation by polyploid giant cancer cells (PGCCs). BMC Cancer. 2022; 22: 410. 35421971 10.1186/s12885-022-09503-6 PMC9012005

[35] Hitz F, Kraus M, Pabst T, Hess D, Besse L, Silzle T, Nelfinavir and lenalidomide/dexamethasone in patients with lenalidomide-refractory multiple myeloma. A phase I/II Trial (SAKK 39/10). Blood Cancer Journal. 2019; 9: 70. 31455773 10.1038/s41408-019-0228-2 PMC6711992

[36] McLean K, VanDeVen NA, Sorenson DR, Daudi S, Liu JR. The HIV protease inhibitor saquinavir induces endoplasmic reticulum stress, autophagy, and apoptosis in ovarian cancer cells. Gynecologic Oncology. 2009; 112: 623–630. 19147209 10.1016/j.ygyno.2008.11.028

[37] Tian J, Liu R, Qu Q. Role of endoplasmic reticulum stress on cisplatin resistance in ovarian carcinoma. Oncology Letters. 2017; 13: 1437–1443. 28454274 10.3892/ol.2017.5580 PMC5403381

[38] Park S, Auyeung A, Lee DL, Lambert PF, Carchman EH, Sherer NM. HIV-1 Protease Inhibitors Slow HPV16-Driven Cell Proliferation through Targeted Depletion of Viral E6 and E7 Oncoproteins. Cancers. 2021; 13: 949. 33668328 10.3390/cancers13050949 PMC7956332

[39] Wu Y, Chen Y, Yan X, Dai X, Liao Y, Yuan J, Lopinavir enhances anoikis by remodeling autophagy in a circRNA-dependent manner. Autophagy. 2024; 1–22. 10.1080/15548627.2024.2325304 PMC11210930 38433354

[40] Basile MS, Mazzon E, Krajnovic T, Draca D, Cavalli E, Al-Abed Y, Anticancer and Differentiation Properties of the Nitric Oxide Derivative of Lopinavir in Human Glioblastoma Cells. Molecules (Basel, Switzerland). 2018; 23: 2463. 30261624 10.3390/molecules23102463 PMC6222694

[41] Paskas S, Mazzon E, Basile MS, Cavalli E, Al-Abed Y, He M, Lopinavir-NO, a nitric oxide-releasing HIV protease inhibitor, suppresses the growth of melanoma cells in vitro and in vivo. Investigational New Drugs. 2019; 37: 1014–1028. 30706336 10.1007/s10637-019-00733-3

[42] You J, He Z, Chen L, Deng G, Liu W, Qin L, CH05-10, a novel indinavir analog, is a broad-spectrum antitumor agent that induces cell cycle arrest, apoptosis, endoplasmic reticulum stress and autophagy. Cancer Science. 2010; 101: 2644–2651. 20946116 10.1111/j.1349-7006.2010.01724.x PMC11158428

[43] Rauschenbach L, Wieland A, Reinartz R, Kebir S, Till A, Darkwah Oppong M, Drug repositioning of antiretroviral ritonavir for combinatorial therapy in glioblastoma. European Journal of Cancer (Oxford, England: 1990). 2020; 140: 130–139. 33091717 10.1016/j.ejca.2020.09.017

[44] Rinaldi R, Miglionico R, Nigro I, D’Orsi R, Chiummiento L, Funicello M, Two Novel Precursors of the HIV-1 Protease Inhibitor Darunavir Target the UPR/Proteasome System in Human Hepatocellular Carcinoma Cell Line HepG2. Cells. 2021; 10: 3052. 34831275 10.3390/cells10113052 PMC8618555

[45] Bellisai C, Sciamanna I, Rovella P, Giovannini D, Baranzini M, Pugliese GM, Reverse transcriptase inhibitors promote the remodelling of nuclear architecture and induce autophagy in prostate cancer cells. Cancer Letters. 2020; 478: 133–145. 32112906 10.1016/j.canlet.2020.02.029

[46] Kang BG, Shende M, Inci G, Park SH, Jung JS, Kim SB, Combination of metformin/efavirenz/fluoxetine exhibits profound anticancer activity via a cancer cell-specific ROS amplification. Cancer Biology & Therapy. 2023; 24: 20–32. 36588385 10.1080/15384047.2022.2161803 PMC9809943

[47] Liu W, Song XL, Zhao SC, He M, Wang H, Chen Z, Antitumor Activity and Mechanism of a Reverse Transcriptase Inhibitor, Dapivirine, in Glioblastoma. Journal of Cancer. 2018; 9: 117–128. 29290776 10.7150/jca.21965 PMC5743718

[48] Li C, Zhang Y, Liu J, Kang R, Klionsky DJ, Tang D. Mitochondrial DNA stress triggers autophagy-dependent ferroptotic death. Autophagy. 2021; 17: 948–960. 32186434 10.1080/15548627.2020.1739447 PMC8078708

[49] Patties I, Kortmann RD, Menzel F, Glasow A. Enhanced inhibition of clonogenic survival of human medulloblastoma cells by multimodal treatment with ionizing irradiation, epigenetic modifiers, and differentiation-inducing drugs. Journal of Experimental & Clinical Cancer Research: CR. 2016; 35: 94. 27317342 10.1186/s13046-016-0376-1 PMC4912728

[50] Abouelezz HM, El-Kashef DH, Abdelaziz RR, Nader MA. Tenofovir alone or combined with doxorubicin abrogates DMBA-induced mammary cell carcinoma: An insight into its modulatory impact on oxidative/Notch/apoptotic signaling. Life Sciences. 2023; 326: 121798. 37236603 10.1016/j.lfs.2023.121798

[51] Ly TTG, Yun J, Ha JS, Kim YJ, Jang WB, Van Le TH, Inhibitory Effect of Etravirine, a Non-Nucleoside Reverse Transcriptase Inhibitor, via Anterior Gradient Protein 2 Homolog Degradation against Ovarian Cancer Metastasis. International Journal of Molecular Sciences. 2022; 23: 944. 35055132 10.3390/ijms23020944 PMC8777939

[52] Zhao R, Bei X, Yang B, Wang X, Jiang C, Shi F, Endothelial cells promote metastasis of prostate cancer by enhancing autophagy. Journal of Experimental & Clinical Cancer Research: CR. 2018; 37: 221. 30200999 10.1186/s13046-018-0884-2 PMC6131784

[53] Robertson AG, Kim J, Al-Ahmadie H, Bellmunt J, Guo G, Cherniack AD, Comprehensive Molecular Characterization of Muscle-Invasive Bladder Cancer. Cell. 2018; 174: 1033. 30096301 10.1016/j.cell.2018.07.036 PMC6297116

[54] Maharjan CK, Ear PH, Tran CG, Howe JR, Chandrasekharan C, Quelle DE. Pancreatic Neuroendocrine Tumors: Molecular Mechanisms and Therapeutic Targets. Cancers. 2021; 13: 5117. 34680266 10.3390/cancers13205117 PMC8533967

[55] Viracept (nelfinavir) [package insert]. La Jolla, CA: Agouron Pharmaceuticals, Inc.; 2005.

[56] Merry C, Barry MG, Mulcahy F, Ryan M, Heavey J, Tjia JF, Saquinavir pharmacokinetics alone and in combination with ritonavir in HIV-infected patients. AIDS (London, England). 1997; 11: F29–F33. 9084785 10.1097/00002030-199704000-00001

[57] KALETRA (lopinavir/ritonavir) [package insert]. North Chicago, IL: AbbVie Inc. 2020.

[58] Esposito V, Palescandolo E, Spugnini EP, Montesarchio V, De Luca A, Cardillo I, Evaluation of antitumoral properties of the protease inhibitor indinavir in a murine model of hepatocarcinoma. Clinical Cancer Research: an Official Journal of the American Association for Cancer Research. 2006; 12: 2634–2639. 16638877 10.1158/1078-0432.CCR-05-2188

[59] Norvir (ritonavir) [package insert]. North Chicago, IL: Abbott Laboratories; 2001.

[60] World Health Organization. Update of recommendations on first- and second-line antiretroviral regimens. 2019. Available at: https://www.who.int/publications/i/item/WHO-CDS-HIV-19.15 (Accessed: 15 August 2023).

[61] Landriscina M, Spadafora C, Cignarelli M, Barone C. Antitumor activity of non-nucleosidic reverse transcriptase inhibitors. Current Pharmaceutical Design. 2007; 13: 737–747. 17346188 10.2174/138161207780249191

[62] Shmakova A, Germini D, Vassetzky Y. HIV-1, HAART and cancer: A complex relationship. International Journal of Cancer. 2020; 146: 2666–2679. 31603989 10.1002/ijc.32730

[63] Sciamanna I, Gualtieri A, Cossetti C, Osimo EF, Ferracin M, Macchia G, A tumor-promoting mechanism mediated by retrotransposon-encoded reverse transcriptase is active in human transformed cell lines. Oncotarget. 2013; 4: 2271–2287. 24345856 10.18632/oncotarget.1403 PMC3926826

[64] Sciamanna I, Landriscina M, Pittoggi C, Quirino M, Mearelli C, Beraldi R, Inhibition of endogenous reverse transcriptase antagonizes human tumor growth. Oncogene. 2005; 24: 3923–3931. 15806170 10.1038/sj.onc.1208562

[65] Landriscina M, Fabiano A, Altamura S, Bagalà C, Piscazzi A, Cassano A, Reverse transcriptase inhibitors down-regulate cell proliferation in vitro and in vivo and restore thyrotropin signaling and iodine uptake in human thyroid anaplastic carcinoma. The Journal of Clinical Endocrinology and Metabolism. 2005; 90: 5663–5671. 16030158 10.1210/jc.2005-0367

[66] Hecht M, Erber S, Harrer T, Klinker H, Roth T, Parsch H, Efavirenz Has the Highest Anti-Proliferative Effect of Non-Nucleoside Reverse Transcriptase Inhibitors against Pancreatic Cancer Cells. PloS One. 2015; 10: e0130277. 26086472 10.1371/journal.pone.0130277 PMC4473268

[67] Hecht M, Harrer T, Büttner M, Schwegler M, Erber S, Fietkau R, Cytotoxic effect of efavirenz is selective against cancer cells and associated with the cannabinoid system. AIDS (London, England). 2013; 27: 2031–2040. 23612009 10.1097/QAD.0b013e3283625444

[68] Brüning A, Jückstock J, Kost B, Tsikouras P, Weissenbacher T, Mahner S, Induction of DNA damage and apoptosis in human leukemia cells by efavirenz. Oncology Reports. 2017; 37: 617–621. 27878300 10.3892/or.2016.5243

[69] Patnala R, Lee SH, Dahlstrom JE, Ohms S, Chen L, Dheen ST, Inhibition of LINE-1 retrotransposon-encoded reverse transcriptase modulates the expression of cell differentiation genes in breast cancer cells. Breast Cancer Research and Treatment. 2014; 143: 239–253. 24337508 10.1007/s10549-013-2812-7 PMC3889873

[70] Houédé N, Pulido M, Mourey L, Joly F, Ferrero JM, Bellera C, A phase II trial evaluating the efficacy and safety of efavirenz in metastatic castration-resistant prostate cancer. The Oncologist. 2014; 19: 1227–1228. 25355844 10.1634/theoncologist.2014-0345 PMC4257751

[71] Dou Z, Ivanov A, Adams PD, Berger SL. Mammalian autophagy degrades nuclear constituents in response to tumorigenic stress. Autophagy. 2016; 12: 1416–1417. 26654219 10.1080/15548627.2015.1127465 PMC4968220

[72] Dou Z, Xu C, Donahue G, Shimi T, Pan JA, Zhu J, Autophagy mediates degradation of nuclear lamina. Nature. 2015; 527: 105–109. 26524528 10.1038/nature15548 PMC4824414

[73] Staszewski S, Morales-Ramirez J, Tashima KT, Rachlis A, Skiest D, Stanford J, Efavirenz plus zidovudine and lamivudine, efavirenz plus indinavir, and indinavir plus zidovudine and lamivudine in the treatment of HIV-1 infection in adults. Study 006 Team. The New England Journal of Medicine. 1999; 341: 1865–1873. 10601505 10.1056/NEJM199912163412501

[74] Marzolini C, Telenti A, Decosterd LA, Greub G, Biollaz J, Buclin T. Efavirenz plasma levels can predict treatment failure and central nervous system side effects in HIV-1-infected patients. AIDS (London, England). 2001; 15: 71–75. 11192870 10.1097/00002030-200101050-00011

[75] Burger D, van der Heiden I, la Porte C, van der Ende M, Groeneveld P, Richter C, Interpatient variability in the pharmacokinetics of the HIV non-nucleoside reverse transcriptase inhibitor efavirenz: the effect of gender, race, and CYP2B6 polymorphism. British Journal of Clinical Pharmacology. 2006; 61: 148–154. 16433869 10.1111/j.1365-2125.2005.02536.x PMC1885008

[76] Carr DF, la Porte CJL, Pirmohamed M, Owen A, Cortes CP. Haplotype structure of CYP2B6 and association with plasma efavirenz concentrations in a Chilean HIV cohort. The Journal of Antimicrobial Chemotherapy. 2010; 65: 1889–1893. 20639527 10.1093/jac/dkq260 PMC2920179

[77] Komatsu M, Kurokawa H, Waguri S, Taguchi K, Kobayashi A, Ichimura Y, The selective autophagy substrate p62 activates the stress responsive transcription factor Nrf2 through inactivation of Keap1. Nature Cell Biology. 2010; 12: 213–223. 20173742 10.1038/ncb2021

[78] Jain A, Lamark T, Sjøttem E, Larsen KB, Awuh JA, Øvervatn A, p62/SQSTM1 is a target gene for transcription factor NRF2 and creates a positive feedback loop by inducing anti-oxidant response element-driven gene transcription. The Journal of Biological Chemistry. 2010; 285: 22576–22591. 20452972 10.1074/jbc.M110.118976 PMC2903417

[79] Klionsky DJ, Abdel-Aziz AK, Abdelfatah S, Abdellatif M, Abdoli A, Abel S, Guidelines for the use and interpretation of assays for monitoring autophagy (4th edition)1 . Autophagy. 2021; 17: 1–382. 10.1080/15548627.2020.1797280 PMC7996087 33634751

[80] Kaizuka T, Morishita H, Hama Y, Tsukamoto S, Matsui T, Toyota Y, An Autophagic Flux Probe that Releases an Internal Control. Molecular Cell. 2016; 64: 835–849. 27818143 10.1016/j.molcel.2016.09.037

[81] Galluzzi L, Vitale I, Aaronson SA, Abrams JM, Adam D, Agostinis P, Molecular mechanisms of cell death: recommendations of the Nomenclature Committee on Cell Death 2018. Cell Death and Differentiation. 2018; 25: 486–541. 29362479 10.1038/s41418-017-0012-4 PMC5864239

[82] HIVID (Zalcitabine) [package insert]. Nutley, NJ: Roche Pharmaceuticals. 2000.

[83] Rossi A, Russo G, Puca A, La Montagna R, Caputo M, Mattioli E, The antiretroviral nucleoside analogue Abacavir reduces cell growth and promotes differentiation of human medulloblastoma cells. International Journal of Cancer. 2009; 125: 235–243. 19358275 10.1002/ijc.24331 PMC2782444

[84] Capparelli EV, Letendre SL, Ellis RJ, Patel P, Holland D, Mc-Cutchan JA. Population pharmacokinetics of abacavir in plasma and cerebrospinal fluid. Antimicrobial Agents and Chemotherapy. 2005; 49: 2504–2506. 15917556 10.1128/AAC.49.6.2504-2506.2005 PMC1140502

[85] Havens JP, Podany AT, Scarsi KK, Fletcher CV. Clinical Pharmacokinetics and Pharmacodynamics of Etravirine: An Updated Review. Clinical Pharmacokinetics. 2020; 59: 137–154. 31679131 10.1007/s40262-019-00830-9 PMC7034776

[86] Høyer-Hansen M, Jäättelä M. Connecting endoplasmic reticulum stress to autophagy by unfolded protein response and calcium. Cell Death and Differentiation. 2007; 14: 1576–1582. 17612585 10.1038/sj.cdd.4402200

[87] Kaushik S, Tasset I, Arias E, Pampliega O, Wong E, Martinez-Vicente M, Autophagy and the hallmarks of aging. Ageing Research Reviews. 2021; 72: 101468. 34563704 10.1016/j.arr.2021.101468 PMC8616816

[88] Cuervo AM, Wong E. Chaperone-mediated autophagy: roles in disease and aging. Cell Research. 2014; 24: 92–104. 24281265 10.1038/cr.2013.153 PMC3879702

