# Supplementary material for: Antiretroviral Drugs Impact Autophagy: Opportunities for Drug Repurposing
Source: Front Biosci (Landmark Ed). Author manuscript; Available in PMC 2025 Feb 19. (PMC11837255; doi:10.31083/j.fbl2907242)
Supplement: supplementary tables [file NIHMS2053852-supplement-supplementary_tables.pdf]

Supplementary Material

Search strategy and selection criteria

Publications reviewed herein were selected from papers identified by MEDLINE searches. MeSH terms used were: "cancer" or "neoplasm" or "malignancy" AND "autophagy." These were paired with each of the following terms: "antiretroviral drug," "HIV protease inhibitor," "integrase inhibitor", "reverse transcriptase inhibitor," "Ibalizumab," "Fostemsavir," "Cenicriviroc," "Enfuvirtide," "Lenacapavir," "Zidovudine," "Didanosine," "Zalcitabine," "Stavudine," "Lamivudine," "Abacavir," "Tenofovir," "Emtricitabine," "Dapivirine," "Nevirapine," "Delavirdine," "Efavirenz," "Etravirine," "Rilpivirine," "Doravirine," Islatravir," "Raltegravir," "Elvitegravir," "Dolutegravir," "Bictegravir," "Cabotegravir," "Saquinavir," "Indinavir," "Nelfinavir," "Ritonavir," "Lopinavir," "Amprenavir," "Atazanavir," "Fosamprenavir," "Tipranavir," or "Darunavir." Articles chosen for inclusion were those that performed any experiment to evaluate autophagy after treatment with an antiretroviral drug or an analog of an antiretroviral drug in the context specifically of a cancer model, unless the drug or analog was used for direct antiviral effects such as in malignancies related to Acquired Immunodeficiency Syndrome, Human T-lymphotropic virus-1, Human Herpesvirus 8, Epstein-Barr virus, or Hepatitis B Virus. Other references were chosen for relevancy to the immediate topic. Only papers published in the English language were included.

Supplemental Table 1. Examples of the anti-tumor effects of antiretroviral drugs.

Antiretroviral drug	Effects leading to cancer cell death	Ref.
Entry Inhibitors		
Cenicriviroc	Inhibits chemokine signaling; inhibits angiogenesis	[1-3]
Ibalizumab (modified)	Inhibits TGF[1]-β	[4]
Maraviroc	Causes cell cycle arrest; reduces macrophage accumulation in tumors; inhibits angiogenesis; chemosensitizer; inhibits CCR5[2] signaling	[2, 5-9]
Rev. Transc. Inhib.		
Abacavir	Decreases cell migration; inhibits cell proliferation; radiosensitizer	[10-12]
Didanosine (modified)	Decreases cell proliferation; causes DNA damage	[13]
Doravirine	Decreases cell migration; causes cell cycle arrest	[14]
Efavirenz	Causes oxidative and mitochondrial stress; radiosensitizer; decreases metabolic activity; causes DNA damage; causes mitochondrial damage	[15-22]
Emtricitabine	Causes DNA damage; causes cell cycle arrest	[23]
Etravirine	Causes cell cycle arrest; causes DNA damage; decreases cell viability	[22, 24]
Lamivudine	Decreases cell migration; inhibits cell proliferation; causes cell cycle arrest; radiosensitizer	[10, 11, 14, 25]
Nevirapine	Decreases cell migration; causes DNA damage	[26, 27]
Rilpivirine	Decreases cell viability; causes cell cycle arrest	[24, 28]
Stavudine	Causes DNA damage; induces oxidative stress; inhibits cell proliferation; inhibits LINE-1[3] retrotransposition	[10, 21, 29]
Tenofovir	Causes DNA damage; causes cell cycle arrest; decreases cell migration; inhibits LINE-1 retrotransposition	[10, 22, 23, 29-31]
Zalcitabine	Radiosensitizer	[32]
Zidovudine	Inhibits DNA polymerase; decreases metabolic activity; causes mitochondrial damage; inhibits LINE-1 retrotransposition chemosensitizer; radiosensitizer	[11, 20, 21, 29, 33, 34]
Integrase Inhibitors		
Cabotegravir	Decreases cell migration	[14]
Dolutegravir (modified)	Perturbs intracellular calcium levels; inhibits cell proliferation	[35, 36]
Elvitegravir	Inhibits metastasis	[37]
Raltegravir	Decreases cell migration	[38]
Protease Inhibitors		
Amprenavir	Inhibits p-AKT[4], ERK1/2[5] signaling	[39, 40]
Atazanavir	Causes ER[6] stress; inhibits cell proliferation	[41, 42]
Darunavir	Decreases cell viability	[24]
Indinavir	Decreases MMP[7] secretion and MMP activity; inhibits angiogenesis; restores T-cell responsiveness	[43-45]
Lopinavir	Causes ER stress; induces cell cycle arrest; inhibits cell proliferation; causes oxidative stress; inhibits cell proliferation; chemosensitizer	[19, 42, 46-48]
Nelfinavir	Inhibits the proteosome; causes ER and mitochondrial stress; causes cell cycle arrest; radiosensitizer; chemosensitizer	[41, 46, 49-53]
Ritonavir	Inhibits the proteosome; causes cell cycle arrest; changes T-cell epitope processing; inhibits angiogenesis; inhibits cell proliferation	[19, 42, 54-56]
Saquinavir	Restores T-cell responsiveness; inhibits the proteosome; inhibits p-AKT; inhibits angiogenesis	[45, 57, 58]
Tipranavir	Chemosensitizer; inhibits cell proliferation	[59, 60]

[1]Transforming growth factor; [2]C-C chemokine receptor type 5; [3]Long interspersed element-1; [4]Protein Kinase B; [5]Extracellular signal-regulated kinase 1/2; [6]Endoplasmic reticulum; [7]Matrix Metalloprotease

Supplemental Table 2. Clinical trials of antiretroviral drugs as chemotherapeutic agents registered at clinicaltrials.gov[1].

Antiretroviral drug	Malignancy	NCT[2] Number	Phase	Status	Accompanying treatments
Entry Inhibitors					
Maraviroc	Colorectal, liver	NCT01736813	Observational	Completed	
	Colorectal, pancreatic	NCT04721301	I	Completed	w/ two biologics
	Colorectal	NCT03274804	I	Completed	w/ pembrolizumab
Rev. Transc. Inhib.					
Efavirenz	Solid tumors or NHL[3]	NCT01878890	I	Completed	
	Prostate	NCT00964002	II	Completed	
	Pancreatic	NCT00964171	II	Unknown	
	Breast	NCT05076682	II	Recruiting	w/CT, anti-PD[12]-1 antibody
Lamivudine	SCLC[4]	NCT04696575	II	Recruiting	w/ platinum-based CT[13]
	p53-mutant colon	NCT03144804	II	Completed	
Zidovudine	Adult T-cell	NCT02737046	II	Recruiting	w/ belinostat, IFN[14]-α-2b
	Adult-T-cell	NCT01941680	Observational	Completed	w/ polyCT, pegylated IFN
	KSHV[5]-associated MCD[6]	NCT00092222	II	Active	
Integrase Inhibitors					
Raltegravir	SCC[7] of head and neck	NCT01275183	Early I	Completed	w/ cisplatin
Protease Inhibitors					
Atazanavir	Solid tumors, hematologic	NCT04184869	Extension	Completed	w/ belinostat
Indinavir	Pediatric sarcoma	NCT00001566	II	Completed	w/ autologous T-cell transplant
Indinavir/Ritonavir	Tumors w/ brain metastases	NCT00637637	II	Unknown	w/ EBRT[15]
Nelfinavir	Solid tumors, or prostate	NCT05036226	I/II	Recruiting	w/ COAST[16]
	Vulvar	NCT04169763	I	Recruiting	w/ cisplatin, EBRT
	MM[8]	NCT03829020	I	Active	w/ bortezomib, metformin
	Solid tumor or lymphoma	NCT03422874	I	Withdrawn	w/ MLN9708
	Cervical	NCT03256916	III	Recruiting	w/ cisplatin, pelvic EBRT
	Melanoma, lung or kidney	NCT03050060	II	Terminated	w/ a biologic, RT[17]
	Cervical	NCT02363829	I	Completed	w/ cisplatin, RT
	Head and neck SCC	NCT02207439	II	Completed	w/ CT, RT
	MM	NCT02188537	II	Completed	w/ bortezomib, dexamethasone
	Pancreatic	NCT02024009	I/II	Unknown	w/ polyCT, RT
	Pancreatic	NCT01959672	II	Completed	w/ polyCT, RT
	CIN[9] 2/3 or CIN3	NCT01925378	II	Withdrawn	
	Progressive MM	NCT01555281	I/II	Terminated	w/ lenalidomide, dexamethasone
	Cervical	NCT01485731	I	Completed	w/ cisplatin, pelvic RT
	NSCLC[10]	NCT01447589	I/II	Withdrawn	w/ radical RT
	Solid tumors	NCT01445106	I	Completed	
	Hematologic	NCT01164709	I	Completed	w/ bortezomib
	NSCLC	NCT01108666	II	Terminated	w/ CT, proton beam RT
	Pancreatic	NCT01086332	I	Terminated	w/ gemcitabine, RT
	Any	NCT01079286	I	Completed	w/ temsirolimus
	Pancreatic	NCT01068327	I	Completed	w/ polyCT, RT
	Adenoid cystic	NCT01065844	II	Completed	
	Glioblastoma	NCT01020292	I	Completed	w/ CT, RT
	Glioblastoma	NCT00915694	I	Terminated	w/ temozolomide, RT
	NSCLC	NCT00791336	II	Terminated	w/ CT, RT
	Rectal	NCT00704600	I/II	Completed	w/ CT, RT
	Glioblastoma	NCT00694837	I	Completed	w/ temozolomide, CT, RT
	NSCLC	NCT00589056	I/II	Completed	w/ polyCT, thoracic RT
	Liposarcoma	NCT00233948	I/II	Terminated	
Ritonavir	Breast	NCT05150691	I/IIa	Recruiting	
	Melanoma, CLL[11]	NCT02948283	I	Completed	w/ metformin
	Glioblastoma	NCT02770378	I/II	Completed	w/ temozolomide, polyCT
	Breast	NCT01009437	I	Completed	
Ritonavir/Lopinavir	Gliomas	NCT01095094	II	Terminated	

[1]This table includes only trials registered at clinicaltrials.gov. It does not include investigator-initiated studies, those not intended for Federal Drug Administration approval or package insert changes, or others that may be self-funded at an institution, by foundations, or by certain government agencies that are not registered at clinicaltrials.gov. This review is focused on the benefits of the off-target effects of antiretroviral drugs; therefore, this table also does not include trials of antiretroviral drugs: 1) when the drug is being used for a direct antiviral effect as in treatment of Acquired

Immunodeficiency Syndrome, Human T-lymphotropic virus-1, Human Herpesvirus 8, Epstein-Barr virus, or Hepatitis B Virus (HBV) related malignancies; 2) for prophylaxis of HBV reactivation in the setting of receiving chemotherapy; 3) when Ritonavir is being used to boost chemotherapy drug levels by its action on cytochrome P450(CYP3A); 4) for pharmacokinetic studies in healthy subjects. [2]National Clinical Trial; [3]Non-Hodgkin's lymphoma; [4]Small cell lung cancer; [5]Kaposi's sarcoma herpesvirus; [6]Multicentric Castleman's disease; [7]Squamous cell carcinoma; [8]Multiple Myeloma; [9]Cervical intraepithelial neoplasia; [10]Non-small cell lung cancer; [11]Chronic lymphocytic leukemia; [12]Programmed death protein 1; [13]Chemotherapy; [14]Interferon; [15]External beam radiation therapy; [16]Combination of autophagy selective therapeutics; [17]Radiation therapy

References for Supplemental Table 1

[1] Eslami M, Azizi Jalilian F, Najafi R, Mahdavinezhad A, Amini R. Promising Modulatory Effects of Cenicriviroc on the Progression of Mouse Colorectal Cancer through Inhibition of CCR2_CCL2 Signaling Pathway. Evid Based Complement Alternat Med. 2023;2023:5993866.
[2] Zeng H, Hou Y, Zhou X, Lang L, Luo H, Sun Y, et al. Cancer-associated fibroblasts facilitate premetastatic niche formation through lncRNA SNHG5-mediated angiogenesis and vascular permeability in breast cancer. Theranostics. 2022;12:7351-70.
[3] Sugiyama S, Yumimoto K, Fujinuma S, Nakayama KI. Identification of effective CCR2 inhibitors for cancer therapy using humanized mice. J Biochem. 2024;175:195-204.
[4] Li S, Liu M, Do MH, Chou C, Stamatiades EG, Nixon BG, et al. Cancer immunotherapy via targeted TGF-beta signalling blockade in T(H) cells. Nature. 2020;587:121-5.
[5] Huang H, Zepp M, Georges RB, Jarahian M, Kazemi M, Eyol E, et al. The CCR5 antagonist maraviroc causes remission of pancreatic cancer liver metastasis in nude rats based on cell cycle inhibition and apoptosis induction. Cancer Lett. 2020;474:82-93.
[6] Casagrande N, Borghese C, Corona G, Aldinucci D. In ovarian cancer maraviroc potentiates the antitumoral activity and further inhibits the formation of a tumor-promoting microenvironment by trabectedin. Biomed Pharmacother. 2024;172:116296.
[7] Passman AM, Strauss RP, McSpadden SB, Finch-Edmondson M, Andrewartha N, Woo KH, et al. Maraviroc Prevents HCC Development by Suppressing Macrophages and the Liver Progenitor Cell Response in a Murine Chronic Liver Disease Model. Cancers (Basel). 2021;13.
[8] Pervaiz A, Zepp M, Mahmood S, Ali DM, Berger MR, Adwan H. CCR5 blockage by maraviroc: a potential therapeutic option for metastatic breast cancer. Cell Oncol (Dordr). 2019;42:93-106.
[9] Zhao R, Bei X, Yang B, Wang X, Jiang C, Shi F, et al. Endothelial cells promote metastasis of prostate cancer by enhancing autophagy. J Exp Clin Cancer Res. 2018;37:221.
[10] Sekeroglu ZA, Sekeroglu V, Kucuk N. Effects of Reverse Transcriptase Inhibitors on Proliferation, Apoptosis, and Migration in Breast Carcinoma Cells. Int J Toxicol. 2021;40:52-61.
[11] Chen X, Wang C, Guan S, Liu Y, Han L, Cheng Y. Zidovudine, abacavir and lamivudine increase the radiosensitivity of human esophageal squamous cancer cell lines. Oncol Rep. 2016;36:239-46.
[12] Carlini F, Ridolfi B, Molinari A, Parisi C, Bozzuto G, Toccacieli L, et al. The reverse transcription inhibitor abacavir shows anticancer activity in prostate cancer cell lines. PLoS One. 2010;5:e14221.
[13] Thaslim Basha SK, Mahaboob Basha S, Subba Rao D, Rasheed S, Varalakshmi M, Raju CN. Synthesis, in silico and in vitro anti-cancer studies of phosphorylated derivatives of didanosine targeting MDA-MB-231 breast cancer cell lines. J Recept Signal Transduct Res. 2023;43:144-53.
[14] Zanre V, Bellinato F, Cardile A, Passarini C, Monticelli J, Di Bella S, et al. Lamivudine, Doravirine, and Cabotegravir Downregulate the Expression of Human Endogenous Retroviruses (HERVs), Inhibit Cell Growth, and Reduce Invasive Capability in Melanoma Cell Lines. Int J Mol Sci. 2024;25.
[15] Bruning A, Juckstock J, Kost B, Tsikouras P, Weissenbacher T, Mahner S, et al. Induction of DNA damage and apoptosis in human leukemia cells by efavirenz. Oncol Rep. 2017;37:617-21.

[16] Hecht M, Erber S, Harrer T, Klinker H, Roth T, Parsch H, et al. Efavirenz Has the Highest Anti-Proliferative Effect of Non-Nucleoside Reverse Transcriptase Inhibitors against Pancreatic Cancer Cells. PLoS One. 2015;10:e0130277.
[17] Hecht M, Harrer T, Korber V, Sarpong EO, Moser F, Fiebig N, et al. Cytotoxic effect of Efavirenz in BxPC-3 pancreatic cancer cells is based on oxidative stress and is synergistic with ionizing radiation. Oncol Lett. 2018;15:1728-36.
[18] Marima R, Hull R, Dlamini Z, Penny C. Efavirenz induces DNA damage response pathway in lung cancer. Oncotarget. 2020;11:3737-48.
[19] Marima R, Hull R, Dlamini Z, Penny C. Efavirenz and Lopinavir/Ritonavir Alter Cell Cycle Regulation in Lung Cancer. Front Oncol. 2020;10:1693.
[20] Schneider MA, Buzdin AA, Weber A, Clavien PA, Borger P. Combination of Antiretroviral Drugs Zidovudine and Efavirenz Impairs Tumor Growths in a Mouse Model of Cancer. Viruses. 2021;13.
[21] Fattore L, Malpicci D, Milite C, Castellano S, Sbardella G, Botti G, et al. Reverse transcriptase inhibition potentiates target therapy in BRAF-mutant melanomas: effects on cell proliferation, apoptosis, DNA-damage, ROS induction and mitochondrial membrane depolarization. Cell Commun Signal. 2020;18:150.
[22] Perna A, Lucariello A, Sellitto C, Agliata I, Carleo MA, Sangiovanni V, et al. Different Cell Cycle Modulation in SKOV-3 Ovarian Cancer Cell Line by Anti-HIV Drugs. Oncol Res. 2017;25:1617-24.
[23] Bruning A, Burger P, Gingelmaier A, Mylonas I. The HIV reverse transcriptase inhibitor tenofovir induces cell cycle arrest in human cancer cells. Invest New Drugs. 2012;30:1389-95.
[24] Pereira M, Vale N. Exploring Darunavir, Rilpivirine and Etravirine as Potential Therapies for Bladder Cancer: Efficacy and Synergistic Effects. Biomedicines. 2024;12.
[25] Rajurkar M, Parikh AR, Solovyov A, You E, Kulkarni AS, Chu C, et al. Reverse Transcriptase Inhibition Disrupts Repeat Element Life Cycle in Colorectal Cancer. Cancer Discov. 2022;12:1462-81.
[26] Shang H, Zhao J, Yao J, Wang H, Wang S, Dong J, et al. Nevirapine inhibits migration and invasion in dedifferentiated thyroid cancer cells. Thorac Cancer. 2019;10:2243-52.
[27] Stefanidis K, Loutradis D, Vassiliou LV, Anastasiadou V, Kiapekou E, Nikas V, et al. Nevirapine induces growth arrest and premature senescence in human cervical carcinoma cells. Gynecol Oncol. 2008;111:344-9.
[28] Islam S, Teo T, Kumarasiri M, Slater M, Martin JH, Wang S, et al. Combined In Silico and In Vitro Evidence Supporting an Aurora A Kinase Inhibitory Role of the Anti-Viral Drug Rilpivirine and an Anti-Proliferative Influence on Cancer Cells. Pharmaceuticals (Basel). 2022;15.
[29] Jones RB, Garrison KE, Wong JC, Duan EH, Nixon DF, Ostrowski MA. Nucleoside analogue reverse transcriptase inhibitors differentially inhibit human LINE-1 retrotransposition. PLoS One. 2008;3:e1547.
[30] Sherif DA, Makled MN, Suddek GM. The HIV reverse transcriptase Inhibitor Tenofovir suppressed DMH/HFD-induced colorectal cancer in Wistar rats. Fundam Clin Pharmacol. 2021;35:940-54.
[31] Abouelezz HM, El-Kashef DH, Abdsmall ie CRR, Nader MA. Tenofovir alone or combined with doxorubicin abrogates DMBA-induced mammary cell carcinoma: An insight into its modulatory impact on oxidative/Notch/apoptotic signaling. Life Sci. 2023;326:121798.
[32] Coucke PA, Li YX, Copaceanu ML, Paschoud N, Cottin E, Ozsahin M, et al. Cell line specific radiosensitizing effect of zalcitabine (2',3'-dideoxycytidine). Acta Oncol. 1997;36:199-205.
[33] Namba T, Kodama R, Moritomo S, Hoshino T, Mizushima T. Zidovudine, an anti-viral drug, resensitizes gemcitabine-resistant pancreatic cancer cells to gemcitabine by inhibition of the Akt-GSK3beta-Snail pathway. Cell Death Dis. 2015;6:e1795.
[34] McIntyre RL, Molenaars M, Schomakers BV, Gao AW, Kamble R, Jongejan A, et al. Anti-retroviral treatment with zidovudine alters pyrimidine metabolism, reduces translation, and extends healthy longevity via ATF-4. Cell Rep. 2023;42:111928.
[35] Wang WJ, Mao LF, Lai HL, Wang YW, Jiang ZB, Li W, et al. Dolutegravir derivative inhibits proliferation and induces apoptosis of non-small cell lung cancer cells via calcium signaling pathway. Pharmacol Res. 2020;161:105129.

[36] Li J, Lin J, Lin JR, Farris M, Robbins L, Andrada L, et al. Dolutegravir Inhibits Proliferation and Motility of BT-20 Tumor Cells Through Inhibition of Human Endogenous Retrovirus Type K. Cureus. 2022;14:e26525.
[37] Liao L, He Y, Li SJ, Zhang GG, Yu W, Yang J, et al. Anti-HIV Drug Elvitegravir Suppresses Cancer Metastasis via Increased Proteasomal Degradation of m6A Methyltransferase METTL3. Cancer Res. 2022;82:2444-57.
[38] Alburquerque-Gonzalez B, Bernabe-Garcia A, Bernabe-Garcia M, Ruiz-Sanz J, Lopez-Calderon FF, Gonnelli L, et al. The FDA-Approved Antiviral Raltegravir Inhibits Fascin1-Dependent Invasion of Colorectal Tumor Cells In Vitro and In Vivo. Cancers (Basel). 2021;13.
[39] Jiang W, Li X, Li T, Wang H, Shi W, Qi P, et al. Repositioning of amprenavir as a novel extracellular signal-regulated kinase-2 inhibitor and apoptosis inducer in MCF-7 human breast cancer. Int J Oncol. 2017;50:823-34.
[40] Gupta AK, Cerniglia GJ, Mick R, McKenna WG, Muschel RJ. HIV protease inhibitors block Akt signaling and radiosensitize tumor cells both in vitro and in vivo. Cancer Res. 2005;65:8256-65.
[41] Pyrko P, Kardosh A, Wang W, Xiong W, Schonthal AH, Chen TC. HIV-1 protease inhibitors nelfinavir and atazanavir induce malignant glioma death by triggering endoplasmic reticulum stress. Cancer Res. 2007;67:10920-8.
[42] Maze EA, Agit B, Reeves S, Hilton DA, Parkinson DB, Laraba L, et al. Human Endogenous Retrovirus Type K Promotes Proliferation and Confers Sensitivity to Antiretroviral Drugs in Merlin-Negative Schwannoma and Meningioma. Cancer Res. 2022;82:235-47.
[43] Esposito V, Palescandolo E, Spugnini EP, Montesarchio V, De Luca A, Cardillo I, et al. Evaluation of antitumoral properties of the protease inhibitor indinavir in a murine model of hepatocarcinoma. Clin Cancer Res. 2006;12:2634-9.
[44] Sharma S, Baksi R, Agarwal M. Repositioning of anti-viral drugs as therapy for cervical cancer. Pharmacol Rep. 2016;68:983-9.
[45] Gruber A, Wheat JC, Kuhen KL, Looney DJ, Wong-Staal F. Differential effects of HIV-1 protease inhibitors on dendritic cell immunophenotype and function. J Biol Chem. 2001;276:47840-3.
[46] Kim JY, Park YJ, Lee BM, Yoon S. Co-treatment With HIV Protease Inhibitor Nelfinavir Greatly Increases Late-phase Apoptosis of Drug-resistant KBV20C Cancer Cells Independently of P-Glycoprotein Inhibition. Anticancer Res. 2019;39:3757-65.
[47] Hampson L, Kitchener HC, Hampson IN. Specific HIV protease inhibitors inhibit the ability of HPV16 E6 to degrade p53 and selectively kill E6-dependent cervical carcinoma cells in vitro. Antivir Ther. 2006;11:813-25.
[48] Paskas S, Mazzon E, Basile MS, Cavalli E, Al-Abed Y, He M, et al. Lopinavir-NO, a nitric oxide-releasing HIV protease inhibitor, suppresses the growth of melanoma cells in vitro and in vivo. Invest New Drugs. 2019;37:1014-28.
[49] Gupta AK, Li B, Cerniglia GJ, Ahmed MS, Hahn SM, Maity A. The HIV protease inhibitor nelfinavir downregulates Akt phosphorylation by inhibiting proteasomal activity and inducing the unfolded protein response. Neoplasia. 2007;9:271-8.
[50] Kawabata S, Gills JJ, Mercado-Matos JR, Lopiccolo J, Wilson W, 3rd, Hollander MC, et al. Synergistic effects of nelfinavir and bortezomib on proteotoxic death of NSCLC and multiple myeloma cells. Cell Death Dis. 2012;3:e353.
[51] Bruning A, Burger P, Vogel M, Rahmeh M, Gingelmaiers A, Friese K, et al. Nelfinavir induces the unfolded protein response in ovarian cancer cells, resulting in ER vacuolization, cell cycle retardation and apoptosis. Cancer Biol Ther. 2009;8:226-32.
[52] Gills JJ, Lopiccolo J, Tsurutani J, Shoemaker RH, Best CJ, Abu-Asab MS, et al. Nelfinavir, A lead HIV protease inhibitor, is a broad-spectrum, anticancer agent that induces endoplasmic reticulum stress, autophagy, and apoptosis in vitro and in vivo. Clin Cancer Res. 2007;13:5183-94.
[53] Koltai T. Nelfinavir and other protease inhibitors in cancer: mechanisms involved in anticancer activity. F1000Res. 2015;4:9.

[54] Srirangam A, Mitra R, Wang M, Gorski JC, Badve S, Baldridge L, et al. Effects of HIV protease inhibitor ritonavir on Akt-regulated cell proliferation in breast cancer. Clin Cancer Res. 2006;12:1883-96.
[55] Lu W, Andrieu JM. HIV protease inhibitors restore impaired T-cell proliferative response in vivo and in vitro: a viral-suppression-independent mechanism. Blood. 2000;96:250-8.
[56] Zhong DS, Lu XH, Conklin BS, Lin PH, Lumsden AB, Yao Q, et al. HIV protease inhibitor ritonavir induces cytotoxicity of human endothelial cells. Arterioscler Thromb Vasc Biol. 2002;22:1560-6.
[57] Pajonk F, Himmelsbach J, Riess K, Sommer A, McBride WH. The human immunodeficiency virus (HIV)-1 protease inhibitor saquinavir inhibits proteasome function and causes apoptosis and radiosensitization in non-HIV-associated human cancer cells. Cancer Res. 2002;62:5230-5.
[58] Sgadari C, Barillari G, Toschi E, Carlei D, Bacigalupo I, Baccarini S, et al. HIV protease inhibitors are potent anti-angiogenic molecules and promote regression of Kaposi sarcoma. Nat Med. 2002;8:225-32.
[59] Xiong JX, Li YT, Tan XY, Chen T, Liu BH, Fu L. Targeting PRSS23 with tipranavir induces gastric cancer stem cell apoptosis and inhibits growth of gastric cancer via the MKK3/p38 MAPK-IL24 pathway. Acta Pharmacol Sin. 2024;45:405-21.
[60] Shen Y, Zhang Q, Zhang L, Wang J, Shu M, Huang K, et al. Virtual screening identifies tipranavir as a SIRT1 inhibitor with anti-hepatocarcinoma effect. Future Med Chem. 2023;15:437-51.
